# Breaking the Pneumatic Barrier: Optimal Control of Mechanically Actuated 2D Grids for High-Throughput, Energy-Efficient Sensor-Based Sorting

**DOI:** 10.3390/s26217030

**Published:** 2026-11-06

**Authors:** Marcel Reith-Braun, Felix Kronenwett, Preetham Suresh, Georg Maier, Harald Kruggel-Emden, Thomas Längle, Jürgen Beyerer, Florian Pfaff, Uwe D. Hanebeck

**Affiliations:** 1 Intelligent Sensor-Actuator-Systems Laboratory, Karlsruhe Institute of Technology, 76131 Karlsruhe, Germany; uwe.hanebeck@kit.edu; 2 Fraunhofer Institute of Optronics, System Technologies and Image Exploitation IOSB, 76131 Karlsruhe, Germany; felix.kronenwett@iosb.fraunhofer.de (F.K.); georg.maier@iosb.fraunhofer.de (G.M.); thomas.laengle@iosb.fraunhofer.de (T.L.); juergen.beyerer@iosb.fraunhofer.de (J.B.); 3 Chair of Mechanical Process Engineering and Solids Processing, Technische Universität Berlin, 10587 Berlin, Germany; preetham.suresh@tu-berlin.de (P.S.); kruggel-emden@tu-berlin.de (H.K.-E.); 4 Institute of Industrial Automation and Software Engineering, University of Stuttgart, 70569 Stuttgart, Germany; florian.pfaff@ias.uni-stuttgart.de

**Keywords:** sensor-based sorting, mechanical actuation, compressed-air-free sorting, two-dimensional grid, real-time model predictive control, optimal control, particle tracking, energy efficiency, recycling technology, industrial automation

## Abstract

Pneumatic ejection has dominated sensor-based sorting for decades, yet diffuse air jets cause bycatch at high mass throughput, and compressed-air generation accounts for a large share of operating cost. We propose (i) a grid-based sorting architecture replacing the conventional single nozzle bar with a two-dimensional grid, and (ii) impact-based mechanical actuators within the grid. Together, these eliminate compressed air entirely while increasing spatio-temporal resolution by leveraging redundant particle–actuator assignments. To coordinate online ejection—deciding which actuators to trigger and when—we formulate sorting as a finite-horizon stochastic optimal control (SOC) problem and introduce mixed discrete–continuous-time control with rollout (MTCR). MTCR integrates open-loop feedback SOC, mixed discrete–continuous-time modeling, situation-aware action-space reduction, and a greedy rollout heuristic, supported by analytically tractable evaluation of expected sorting costs. In high-mass-flow simulations with equal accept/reject ratios, MTCR maintains both true negative rate and true positive rate above 90%, demonstrating effective actuator coordination and bycatch reduction. Laboratory experiments on a 56-solenoid prototype show that the grid significantly exceeds a state-of-the-art pneumatic chute sorter in rejection performance at matched bycatch level, with actuation energy being at least 79 times lower. Grid-based mechanical sorting thus offers a promising route toward precise, energy-efficient separation for cost-sensitive industries such as recycling.

## 1. Introduction

In sensor-based sorting, separation is typically achieved by bursts of compressed air that deflect identified particles from the main particle stream. Conventional sorters guide particles via a belt or chute past a single row of nozzles spanning the belt or chute width, which are triggered to release these air bursts. This pneumatic ejection principle has dominated the field for more than 30 years [1], with only incremental improvements in design and control. Despite its commercial success—largely because it achieves adequate sorting accuracy at economical mass throughput—pneumatic separation suffers from fundamental limitations in accuracy and operating cost. Its main drawbacks are limited temporal and spatial resolution and high energy consumption. Diffuse, expanding air cones reduce separation precision, especially at high mass flow, and increase the risk of accidental ejections [2]. Compressed-air generation and distribution account for up to 70% of a sorter’s operating costs [3], while less than one percent of the primary energy is ultimately used to alter particle trajectories [4]. These constraints hinder the economic use of sensor-based sorting in new or cost-sensitive markets and threaten the profitability of existing operations in sectors under severe economic pressure, such as recycling.

To address these challenges, we propose a sensor-based sorter with two central novelties: (i) a two-dimensional grid of actuators for particle ejection, and (ii) mechanical impact-based actuators instead of compressed air. Together, these elements increase sorting resolution, flexibility, and energy efficiency and open a path toward a new generation of high-throughput sensor-based sorting systems.

Our architecture replaces the conventional one-dimensional air nozzle bar with a two-dimensional grid of slightly offset actuator rows. Unlike conventional sorters, which can eject a particle only at a fixed position and time, the grid allows selection of the best-suited actuator based on both lateral position and expected arrival time, substantially increasing spatial resolution and temporal sampling frequency. It also introduces a new degree of freedom: multiple actuators can potentially eject the same particle. This redundancy in particle–actuator assignment enables optimization of the separation accuracy. A combined tracking and control system can therefore choose activations that maximize rejection of undesired particles while minimizing bycatch, based on predicted trajectories of all particles in the separation zone.

The second novelty replaces compressed-air bursts with mechanical impact actuators, e.g., mounted on a chute. These actuators deliver short, high-energy impulses that deflect targeted particles from their trajectories. Eliminating compressed air improves energy efficiency by one to two orders of magnitude as confirmed by our system-level comparison in Section 7.6. While mechanical actuators in particle separation are not new [1,4,5,6], their integration into a two-dimensional grid yields synergistic advantages. Individual mechanical actuators are typically larger than air nozzles and therefore offer coarser inherent spatial resolution, but the grid compensates for this limitation. Because mechanical actuators produce sharply bounded impact zones, the effective spatial resolution can even exceed that of conventional pneumatic sorters. The grid also compensates for the recovery time between consecutive activations, during which the actuator cannot eject a particle. We show that activation delay and pre- and post-impact influence durations are in the range of a few milliseconds, enabling mass flows comparable to state-of-the-art pneumatic sorters.

Exploiting these hardware advantages requires a real-time capable controller that uses the grid’s temporal and spatial redundancy for high-throughput, high-accuracy sorting. On conventional one-dimensional nozzle bars, industrial practice largely assigns each particle to a single nozzle, mostly via simple delay rules [7], although more sophisticated methods—such as predictive tracking [7,8] for particle tracking and precise activation timing, and uncertainty-aware timing schemes [9]—have proven superior. Such decoupled rules do not exhaust the grid’s combinatorial degrees of freedom and cannot resolve conflicts when several reject particles compete for the same actuator or when an activation risks bycatch among accept particles. Online control is therefore significantly harder than in conventional single-row pneumatic sorters.

A principled alternative is to cast sorting as finite-horizon stochastic optimal control (SOC) over predicted particle trajectories and actuator motion, directly encoding minimization of falsely sorted particles as an optimization problem. With typical image rates of 100 fps to 250 fps and a large (≫10) number of actuators to coordinate, the resulting problem combines a demanding real-time scheduling task with partial observability, hard contact-induced switching, and an enormous action space. State-of-the-art sampling-based, often iterative, solvers for stochastic nonlinear model predictive control (SNMPC) are therefore not viable at the required frame rates: candidate-generation and reweighting schemes such as cross-entropy method (CEM) [10,11] and model predictive path integral (MPPI) [12,13] require many candidate sequences in large action spaces and thus too much computation per control step. The same applies to finite action-space solvers such as point-based value iteration (PBVI) [14,15,16] and Monte Carlo tree search (MCTS) [17,18], which struggle with the combinatorial complexity of the action space. Gradient-based local methods for continuous action spaces, such as iterative linear quadratic Gaussian (LQG) [19,20] and differential dynamic programming (DDP) [21], are likewise ill-suited because the actuator–particle interaction is discontinuous: small timing variations can switch the system between qualitatively different outcomes (e.g., no-hit vs. hit vs. bycatch).

We therefore introduce the mixed discrete–continuous-time control with rollout (MTCR) controller as an approximate, suboptimal solution to the sorting problem. It assigns each reject particle to at most one actuator, with activation timed to the predicted arrival time, and combines this with situation-aware action-space pruning to reduce the action space to a finite, manageable size. A mixed discrete–continuous-time system model still allows precise activation at intermediate times despite coarse discretization. Furthermore, a combined limited look-ahead–rollout approach [22,23,24] exhaustively evaluates activations only in the first few stages and assigns the remaining particles by a greedy heuristic. Expected sorting costs are evaluated under an open-loop feedback (OLF) formulation with analytically tractable approximations, avoiding expensive Monte Carlo simulation. This design trades full search-space evaluation for computational speed and remains easy to interpret while improving over purely heuristic assignments.

**Figure 1:**
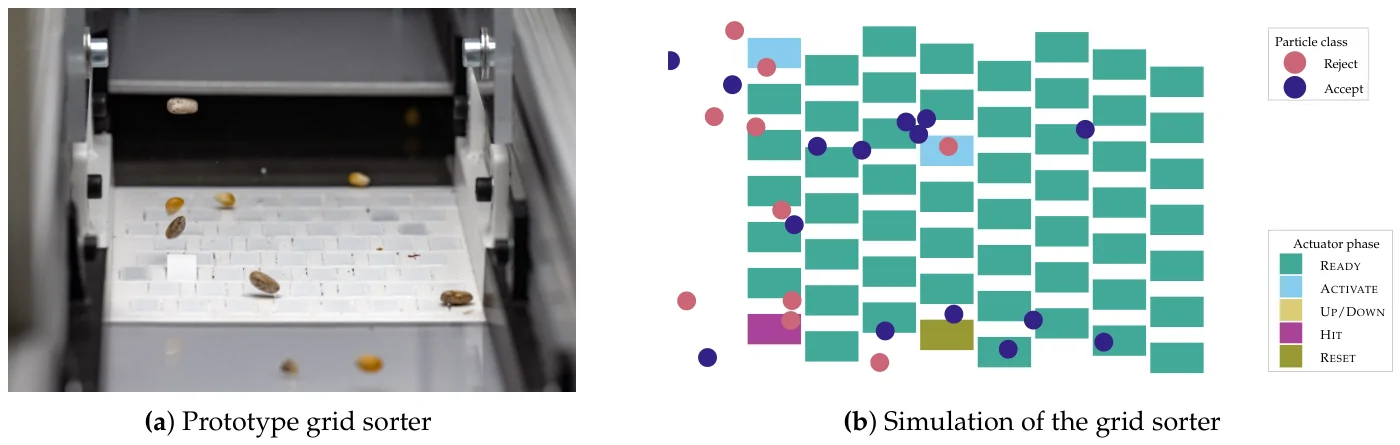
(**a**) Our prototype grid sorter in operation: Particles slide down the chute (from bottom to top), passing a grid of 8 slightly offset rows with 7 mechanical actuators each. Undesired particles are ejected upward and separated from the non-ejected particles by the separating plate (upper part of the figure). (**b**) Our simulation of the prototype grid sorter: Particles move from left to right over actuators that can be in six different phases (see Section 5.2.2 for a detailed explanation). Depending on the phase, particles are ejected with different probabilities. The displayed mass flow corresponds to the “high mass flow” scenario in our evaluation (Section 7).

Our contributions are threefold: (i) we propose a grid-based sorting architecture that replaces the conventional single nozzle bar with a two-dimensional grid as introduced in our first novelty; (ii) we formulate sorting as a finite-horizon SOC problem and introduce the MTCR controller for real-time SNMPC of the grid sorter; and (iii) we design and build a prototype realizing an advantageous special case of the grid architecture—impact-based mechanical actuators on a chute that entirely eliminate compressed air (Figure 1a)—as introduced in our second novelty. We evaluate the MTCR controller and a greedy assignment heuristic in high-mass-flow simulations (see Figure 1b), showing that MTCR consistently outperforms the heuristic. Finally, we evaluate the joint system in laboratory experiments, showing that the prototype already exceeds a conventional high-precision chute sorter in rejection performance at the same bycatch level under the heuristic, while MTCR improves bycatch avoidance on the same grid hardware. Additionally, we measure the grid’s separation energy and establish a lower bound for the pneumatic reference, demonstrating a reduction in separation energy of nearly two orders of magnitude.

Throughout this article, vectors will be indicated by underlined letters, e.g., $\underline{x}$, boldface letters, such as x, will represent random variables, and boldface capital letters, e.g., A, will indicate matrices. As a shorthand notation, we write x∈A if we mean that the set A is the sample space of x. We denote the expectation of a random variable x by $E\left\{x\right\}$ and its variance by $\mathrm{Var}\left\{x\right\}$. Analogously, the covariance of a random vector $\underline{x}$ is denoted by $\mathrm{Cov}\left\{\underline{x}\right\}$. To label a measurement, i.e., a realization, of x, we write $\overset{★}{x}$. To denote a sequence over *n* time steps starting at time step *k*, we will use the index k,0:n. For instance, $x_{k,0:n}$ denotes the sequence $x_{k},\ldots ,x_{k+n}$. Accordingly, index k,n refers to time step k+n.

## 2. Problem Statement

We formulate the control task as a discrete-time, finite-horizon SOC problem with imperfect state feedback. We consider the general nonlinear stochastic system and measurement equations

(1) $$\begin{array}{cc} {\underline{x}}_{k+1} & ={\underline{f}}_{k}\left({\underline{x}}_{k},{\underline{u}}_{k},{\underline{w}}_{k}\right)\;, \end{array}$$

(2) $$\begin{array}{cc} {\underline{z}}_{k} & ={\underline{h}}_{k}\left({\underline{x}}_{k},{\underline{v}}_{k}\right)\;, \end{array}$$

where ${\underline{x}}_{k}\in X_{k}$ and ${\underline{z}}_{k}\in Z_{k}$ are random vectors denoting the system’s state and measurements, respectively, at time step *k*, ${\underline{u}}_{k}\in A_{k}$ is the control input, or action, and ${\underline{w}}_{k}\in W_{k}$ and ${\underline{v}}_{k}\in V_{k}$ are random disturbances. The state ${\underline{x}}_{k}$ is not directly accessible, i.e., we can only gain information about the system state via measurements ${\overset{★}{\underline{z}}}_{k}$ of ${\underline{z}}_{k}$, and by assuming that the initial state is distributed according to ${\underline{x}}_{0}∼p\;\left({\underline{x}}_{0}\right)$.

Let us introduce the information set $I_{k}$ defined as $I_{k}=\left\{p\;\left({\underline{x}}_{0}\right),{\overset{★}{\underline{z}}}_{0:k},{\underline{u}}_{0:k-1}\right\}$ for k>0, and $I_{0}=\left\{p\;\left({\underline{x}}_{0}\right),{\overset{★}{\underline{z}}}_{0}\right\}$. The goal of SOC is to find a sequence of closed-loop feedback (CLF) control laws, or policies, ${\underline{\mu }}_{k,0:N-1}^{\mathrm{CLF}}$, with ${\underline{\mu }}_{k,n}^{\mathrm{CLF}}\left(I_{k,n}\right)\in A_{k,n}$, that map $I_{k,n}$ to actions ${\underline{u}}_{k,n}={\underline{\mu }}_{k,n}^{\mathrm{CLF}}\left(I_{k,n}\right)$ such that the expected cumulative cost

(3) $$\begin{array}{c} J_{k}=E\;\left\{g_{k,N}\left({\underline{x}}_{k,N}\right)+\sum_{n=0}^{N-1}g_{k,n}\left({\underline{x}}_{k,n},{\underline{\mu }}_{k,n}^{\mathrm{CLF}}\left(I_{k,n}\right),{\underline{w}}_{k,n}\right)|I_{k}\right\} \end{array}$$

subject to Equations (1) and (2) is minimized. Here, N∈N denotes the finite control horizon, $g_{k,n}:X_{k,n}\times A_{k,n}\times W_{k,n}\to R$ denotes the one-step objective function as a function of a state–action–disturbance tuple, and $g_{k,N}:X_{k}\to R$ is the terminal objective function. A significant simplification of the control problem is to not consider that future measurements are dependent on the control decisions within the control horizon. In this case, we instead seek a sequence of OLF policies ${\underline{\mu }}_{k,0:N-1}^{\mathrm{OLF}}$, with ${\underline{\mu }}_{k,n}^{\mathrm{OLF}}\left({\tilde{I}}_{k,n}\right)\in A_{k,n}$, mapping the reduced information set ${\tilde{I}}_{k,n}=I_{k}\cup \left\{{\underline{u}}_{k,0:n-1}\right\}$ for n>0 and ${\tilde{I}}_{k,0}=I_{k}$ to actions that minimize Equation (3) under this restricted information structure. Thus, for stages n>0, it is sufficient to predict the system states using Equation (1), at the cost of typically overestimating uncertainty [25]. In model predictive control (MPC), also called receding-horizon control, the optimal control found for the current time step ${\underline{u}}_{k,0}^{*}$ is applied to the system, and the procedure is repeated at time step k+1.

## 3. Related Work

### 3.1. State of the Art in Sensor-Based Sorting

Recent advances in sensor-based sorting increasingly exploit data-driven methods, in particular for particle detection [26,27]. Sorting accuracy can be improved by predictive tracking: particles are tracked by a Kalman filter-based multitarget tracker in an area-scan camera’s field of view (FOV), and the resulting motion estimates are used to forecast arrival times and locations at the nozzles [7,8]. This idea has been extended to particle-specific nozzle activations that account for prediction uncertainty and has been shown to further improve sorting precision [9,28]. In that work, particle arrival was modeled as a first-passage time problem [29,30], and a Gaussian approximation termed the Gauß–Taylor approximation was developed for the first-passage time distribution (FPTD) and for the distribution of the lateral position at arrival. Because it relies on a first-order Taylor expansion, the approximation is linear in the state and therefore computationally convenient. Although it neglects the skewness of the FPTD, it has been shown empirically to be accurate for the small initial and process uncertainties typically encountered in particle tracking for sensor-based sorting [28].

In ref. [31], learned neural network models were combined with hand-crafted motion models in a mixture-of-experts framework, yielding precise and robust trajectory predictions. More recently, an unsupervised image-forecasting approach based on a convolutional long short-term memory network was applied to nozzle activation prediction, reducing operator setup and supervision [32]. A comprehensive numerical and experimental study of neural networks for sorting tasks is given in [33]. Discrete-element method–computational fluid dynamics simulations of sensor-based sorters have been reported in [2,34,35,36,37] and show good agreement with experiments [36,37].

Mechanical separation in sensor-based sorting dates back at least to a 1926 patent for an electromagnetic chute discharge system with trap doors [38]. The dominant contemporary design uses a pneumatic piston to actuate a finger-like deflector. This principle is established in food sorting of high-mass, dense products in dusty and heavily soiled environments, such as potatoes and onions. To the authors’ knowledge, the commercial mechanical food sorters available in 2026 include systems by Tomra, Flikweert Vision, DOWNS, and Karevo, which all appear to follow the pneumatic-piston concept; ATM RECYCLINGSYSTEMS offers mechanical solutions for metal recycling. Mechanical separation systems are often restricted to low-throughput applications [1,4], because their typically higher physical inertia makes them less selective than high-speed air valves.

### 3.2. State of the Art in Stochastic Optimal Control

SOC is typically cast as Markov decision process (MDP) (in OLF control) or partially observable Markov decision process (POMDP) (in CLF control) and solved by dynamic programming (DP) [23,39], with exact solutions only in special cases (LQG, small finite-action MDP); practical controllers therefore apply approximations, depending on whether actions are discrete or continuous.

For finite (or finitely discretized) actions, PBVI and sampling-based POMDP solvers in the infinite-horizon limit are particularly popular; they approximate value iteration over belief states [14,15,16,40,41]. A particularly effective suboptimal scheme for combinatorial decision sequences in the finite-horizon setting is rollout [22,23,24,42]: at each stage, one-step (or multi-step) limited-lookahead minimization is combined with a base heuristic that completes the remaining decisions. That is, a full optimization for the best control input ${\underline{u}}_{k,n}^{*}\in A_{k,n}$ is performed for a limited number of stages $n=0,\ldots ,N_{L}$ while costs are computed for the entire horizon using the heuristically chosen control input for $n=N_{L}+1,\ldots ,N$. Rollout algorithms are guaranteed to be not worse than the heuristic alone [22,23,24]. Related limited-horizon tree search, e.g., via MCTS [17,18], also with pruning via search-space restrictions, is widely used when exhaustive enumeration is infeasible or prior knowledge should be exploited [24,43].

For continuous actions, local gradient-based methods (iterative LQG [19,20], DDP [21]) and constrained nonlinear optimization (sequential quadratic programming [44], interior-point [45]) assume smooth or locally smooth dynamics. Sampling-based planners (input inference control [46], MPPI [12,13,47], CEM [10,11]) avoid derivatives by drawing and (iteratively) reweighting or refitting candidate open-loop sequences. While CEM and MPPI are not restricted to smooth continuous actions, they are most naturally applied to them.

## 4. Challenges

The grid sorting application poses the following challenges, which render standard SOC solution approaches intractable:

Large continuous action space. The number of actuators is typically large (≫10), and each actuator can in principle be activated at any time and with arbitrary intensity. This yields a large continuous or finely discretized action space, for which exact DP and numerical approximations (PBVI, MCTS) quickly become intractable.

Large, typically hybrid and variable-size state space. The system state comprises particles and actuators. The number of particles $N_{P}$ (typically $N_{P}\gg 20$) is variable, as particles continuously enter and leave the field of view. Consequently, the state dimension is not known a priori, and the state space is hybrid, combining continuous components (e.g., particle positions and velocities) with discrete ones (e.g., particle classes). This strongly increases the complexity of state propagation, uncertainty representation, and optimization.

Discontinuous switching dynamics. Control actions affect the particle state only upon physical contact with an actuator. There is no smoothly differentiable relationship between actuator activations and the resulting state transitions, which severely limits the usefulness of gradient-based methods such as DDP and iterative LQG. Moreover, sampling-based solvers (CEM, MPPI) may perform poorly because costs depend on sparse contact events rather than on smooth trajectories.

Combinatorial–timing structure. The control problem couples a combinatorial assignment of particles to actuators with the timing of the corresponding activations. The controller must therefore decide both which actuator serves each particle and when to trigger the associated activation, and must thus be capable of solving complex combinatorial problems.

Real-time requirements. The controller must compute a control decision within a fixed, short time budget. Typical frame rates in area-scan camera-based sorting systems range from 100 fps to 250 fps [7,9], leaving only a few milliseconds for optimization at each time step. This rules out sampling-based approaches such as CEM, MPPI, and MCTS that require computationally expensive (iterative) cost evaluation.

## 5. Approximate Optimal Control of the Grid Sorter

This section describes the design of the proposed controller. We first summarize the key ideas, then introduce the system model, explain prediction in that model, discuss cost evaluation, and finally present candidate-sequence generation as well as algorithmic and implementation details.

### 5.1. Key Ideas

The challenges in Section 4 and the controller literature reviewed in Section 3 rule out a direct transfer of general-purpose MPC solvers. Our design therefore combines problem-specific action-space reductions, an OLF information structure, a mixed discrete–continuous-time system model, and rollout-based combinatorial search to remain computationally tractable under real-time constraints. The following subsections introduce the main design choices; together they yield the MTCR controller.

#### 5.1.1. Design Choices on the Action Space

A naïve formulation would treat every actuator as a continuous input channel and allow multiple hits per particle, yielding a high-dimensional, largely continuous action space. We therefore introduce the following assumptions, which retain the grid’s assignment flexibility but significantly reduce the action space:

**Assumption 1** (Reduction of the Action Space)**.**

*1.*  *We consider only actuator activation times as control inputs determined by the SNMPC.*
*2.*  *For each particle–actuator pair, we consider only one actuator activation time, chosen such that the particle is hit at its expected arrival time at a nominal, momentum-maximizing position.*
*3.*  *For each particle to be ejected, at most one actuator is activated.*

Assumption 1 Item 1 reflects that, in practice, it is typically sufficient to construct actuator activation profiles, such as time–voltage profiles for electromechanical actuators, solely by their activation time, e.g., as a step function with fixed voltage and duration. This avoids optimizing higher-dimensional control trajectories online. Assumption 1 Item 2 couples each activation to a single predicted particle arrival time, analogously to predictive tracking in conventional sensor-based sorting but now as part of a joint assignment rather than a per-particle rule. A control sequence ${\underline{u}}_{k,0:N-1}\in C_{k}$ therefore corresponds to a finite set of particle–actuator assignments, and the action space $A_{k,n}$ becomes discrete with time-varying but finite support. Together, Assumption 1 Items 1 and 2 largely decouple the combinatorial assignment problem from the timing problem, the latter being handled by predicting particle arrival times at the actuators. Assumption 1 Item 3 excludes redundant multi-hit sequences on the same particle: a single well-timed impact is typically sufficient, and allowing multiple hits would greatly increase the combinatorics without materially improving separation. Under these assumptions, control sequences are fully described by particle–actuator assignments (but not vice versa), that is, there exists a mapping from particles to actuators that defines a control sequence.

Under Assumption 1 Item 2 alone, any combination of particle–actuator pairs is formally valid, yielding ${\left(2^{N_{P}^{\mathrm{rej}}}\right)}^{N_{A}}$ possible control sequences, where $N_{P}^{\mathrm{rej}}$ is the number of undesired particles and $N_{A}$ the number of actuators. Assumption 1 Item 3 reduces this number to $|C_{k}|={\left(N_{A}+1\right)}^{N_{P}^{\mathrm{rej}}}$ (the +1 term accounts for leaving a particle unassigned). Even then, not all assignments are feasible—for example, when a particle’s trajectory does not pass over an actuator. We exploit this fact later to prune the huge search space.

#### 5.1.2. Open-Loop Feedback Control

We adopt the OLF paradigm and, when evaluating costs, ignore the effect of measurements that would arrive within the control horizon. This is because CLF SNMPC would nest expectations over future measurements and couple control with estimation inside the horizon (Section 2), which is attractive in principle but intractable here: the belief state is high-dimensional, propagation is nonlinear, and extra expectation evaluation over continuous-valued position measurements would require repeated Monte Carlo sampling. As shown later in Section 5.4, OLF allows expected costs to be approximated analytically—without Monte Carlo rollouts—and thus significantly reduces computational effort. Only measurements received at time step *k* are used to update the information set and estimate the system state; track births and deaths are not modeled within the control horizon.

#### 5.1.3. Mixed Discrete–Continuous-Time System Model Formulation

Because actuator activation times are now tied to predicted particle arrival, the system model must process controls that lie in continuous time and generally do not coincide with discrete time steps. This differs from standard discrete-time SOC, which typically assumes piecewise-constant inputs between sampling instants [48] (Chapter 4.3). To avoid coarse temporal discretization, we derive a continuous-time formulation for the relevant model components so that state variables can be evaluated at arbitrary instants. For these components, the discrete-time model is obtained by evaluating the continuous-time model at the sampling instants. Prediction thus alternates between discrete-time propagation to the next time step and continuous-time evaluation at intermediate instants, such as the beginning and end of a planned actuator motion. This mixed discrete–continuous-time treatment avoids resampling the entire SOC problem at millisecond resolution while allowing precise actuator activation.

#### 5.1.4. Optimization

We build an online SNMPC controller that operates in a receding-horizon manner. The core idea follows directly from the action-space assumptions: while Assumption 1 Item 3 reduces complexity, it couples control decisions across time steps—a particle assigned to an actuator in one step cannot be reassigned later. It is therefore convenient to generate complete candidate control sequences ${\left\{{\underline{u}}_{k,0:N-1}^{\left(i\right)}\right\}}_{i=1,2,\ldots }$ satisfying Assumption 1 Item 3 and then select the best sequence ${\underline{u}}_{k,0:N-1}^{*}$, i.e., we seek

$$\begin{array}{c} {\underline{u}}_{k,0:N-1}^{*}=\underset{{\underline{u}}_{k,0:N-1}\in {\left\{{\underline{u}}_{k,0:N-1}^{\left(i\right)}\right\}}_{i=1,2,\ldots }}{arg min}J_{k}\left({\underline{u}}_{k,0:N-1}\right) \end{array}$$

subject to Equations (1) and (2), with all candidates ${\underline{u}}_{k,0:N-1}^{\left(i\right)}$ satisfying Assumption 1 The control problem then decouples into two steps: (i) generating candidate solutions, and (ii) evaluating the OLF objective for each candidate and applying the sequence with minimum cost. If the candidate set equals the full feasible set $C_{k}$, the method is exact under Assumption 1.

To keep computation tractable, the model (Section 5.2) and cost function (Section 5.4) are designed to permit closed-form evaluation of expected cumulative costs wherever possible, and analytical approximations elsewhere. Because the candidate set remains large, we apply problem-specific pruning and rollout heuristics. Pruning discards assignments that are clearly infeasible or suboptimal, for example, when a particle does not pass over an actuator or an actuator cannot eject a particle given already scheduled activations. Rollouts assign only a subset of the $N_{P}^{\mathrm{rej}}$ reject particles exhaustively and then complete each partial sequence greedily. Both strategies are detailed in Section 5.5.

### 5.2. System and Measurement Model

The MTCR controller relies on a coupled model of particles (Section 5.2.1), actuators (Section 5.2.2), and their contact interaction (Section 5.2.3). At each time step *k*, the state ${\underline{x}}_{k}$ consists of the particle motion states ${\underline{x}}_{k}^{\mathrm{mot}}$, the particle existence indicators ${\mathrm{ex}}_{k}$, and the actuator states ${\underline{a}}_{k}$, that is, ${\underline{x}}_{k}=\left\{{\underline{x}}_{k}^{\mathrm{mot}},{\mathrm{ex}}_{k},{\underline{a}}_{k}\right\}$.

#### 5.2.1. Particle Model

The particle model specifies the motion and existence dynamics, as well as the measurement model. Each particle *i*, $i=1,\ldots ,N_{P}$ with $N_{P}$ being the number of particles, is described by its

- Motion, via the random motion state ${\underline{x}}_{k}^{i,\mathrm{mot}}\in R^{n_{\underline{x}}}$ on the real plane;
- Existence, via the binary random variable ${\mathrm{ex}}_{k}^{i}\in \left\{0,1\right\}$ with ${\mathrm{ex}}_{k}^{i}=1≜$ “particle *i* not yet ejected”;
- Class, via the deterministic binary variable $c^{i}\in \left\{\mathrm{acc},\mathrm{rej}\right\}$ with $c^{i}=\mathrm{rej}≜$ “particle *i* should be removed from the particle stream”.

The class variable $c^{i}$ is viewed as a storage variable only and does not change with time. We furthermore introduce the following set of assumptions.

**Assumption 2**  (Particle Model).

*1.*  *All particles evolve independently and follow the same transition model, that is, particles are mutually independent and identically distributed (i.i.d.).*
*2.*  *The motion of particle i is independent of existence, class, actuator states, and control inputs.*
*3.*  *The particle motion is linear Gaussian with additive noise, i.e., is of the form*
  $$\begin{array}{cc} p\;\left({\underline{x}}_{k}^{i,\mathrm{mot}}|{\underline{x}}_{k-1}^{i,\mathrm{mot}}\right) & =N\left(A{\underline{x}}_{k-1}^{i,\mathrm{mot}},C^{\underline{w}}\right)\;, \end{array}$$
  *with A being the state transition matrix and $C^{\underline{w}}$ the process noise covariance. Additionally, the initial state ${\underline{x}}_{0}^{i,\mathrm{mot}}$ is assumed Gaussian.*
*4.*  *The existence transition of each particle depends on possible contacts with the actuators: It therefore depends on ${\underline{x}}_{k-1}^{i,\mathrm{mot}}$, ${\mathrm{ex}}_{k-1}^{i},{\underline{a}}_{k-1}$ and ${\underline{u}}_{k-1}$.*

While Assumption 2 Item 1 greatly simplifies calculations, it also excludes some physical scenarios, such as particle–particle collisions. By Assumption 2 Items 1, 2 and 4, the transition model decomposes as

(4) $$\begin{array}{cc} p\;\left({\underline{x}}_{k}^{\mathrm{mot}},ex_{k}|{\underline{x}}_{k-1}^{\mathrm{mot}},ex_{k-1},{\underline{a}}_{k-1},{\underline{u}}_{k-1}\right) & =\prod_{i=1}^{N_{P}}p\;\left({\underline{x}}_{k}^{i,\mathrm{mot}}|{\underline{x}}_{k-1}^{i,\mathrm{mot}}\right)\\ \; & \;\;\cdot \;p\;\left(ex_{k}^{i}|{\underline{x}}_{k-1}^{i,\mathrm{mot}},ex_{k-1}^{i},{\underline{a}}_{k-1},{\underline{u}}_{k-1}\right)\;, \end{array}$$

where ${\underline{x}}_{k}^{\mathrm{mot}}={\left[\begin{array}{ccc} {\underline{x}}_{k}^{1,\mathrm{mot}}, & \ldots , & {\underline{x}}_{k}^{N_{P},\mathrm{mot}} \end{array}\right]}^{⊤}$, $ex_{k}={\left[\begin{array}{ccc} ex_{k}^{1}, & \ldots , & ex_{k}^{N_{P}} \end{array}\right]}^{⊤}$. Here, $p\;\left({\underline{x}}_{k}^{i,\mathrm{mot}}|{\underline{x}}_{k-1}^{i,\mathrm{mot}}\right)$ is the motion model and $p\;\left(ex_{k}^{i}|{\underline{x}}_{k-1}^{i,\mathrm{mot}},ex_{k-1}^{i},{\underline{a}}_{k-1},{\underline{u}}_{k-1}\right)$ is the existence model.

We assume time-invariant constant-velocity (CV) or constant-acceleration (CA) motion (see, e.g., [48]), i.e., ${\underline{x}}_{k}^{i,\mathrm{mot}}$ has the form $\left[x,v_{x},y,v_{y}\right]$ (CV) or $\left[x,v_{x},a_{x},y,v_{y},a_{y}\right]$ (CA), where x and y denote position, $v_{x}$ and $v_{y}$ velocity, and $a_{x}$ and $a_{y}$ acceleration in transport (x-) and lateral (y-) direction, respectively. By Assumption 2 Item 4, the existence of each particle evolves as a time-variant hidden Markov chain. For convenience, we introduce the auxiliary random variable ${\mathrm{ej}}_{k}^{i}\in \left\{0,1\right\}$ with ${\mathrm{ej}}_{k}^{i}=1≜\;$“particle *i* ejected at time step *k*” which is determined by the contact and actuator models (Section 5.2.3 and Section 5.2.2). The transition probabilities are then defined as

$$\begin{array}{cc} P\;\left({\mathrm{ex}}_{k}^{i}=0|{\underline{x}}_{k-1}^{i,\mathrm{mot}},ex_{k-1}^{i}=0,{\underline{a}}_{k-1},{\underline{u}}_{k-1}\right) & =1\;,\\ P\;\left({\mathrm{ex}}_{k}^{i}=0|{\underline{x}}_{k-1}^{i,\mathrm{mot}},ex_{k-1}^{i}=1,{\underline{a}}_{k-1},{\underline{u}}_{k-1}\right) & =P\;\left({\mathrm{ej}}_{k-1}^{i}=1|{\underline{x}}_{k-1}^{i,\mathrm{mot}},{\underline{a}}_{k-1},{\underline{u}}_{k-1}\right)\;. \end{array}$$

At each stage n=0, the chain is freshly initialized with $P\;\left({\mathrm{ex}}_{k,0}^{i}=1\right)=1$ for all present particles at time step *k*.

**Assumption 3** (Measurement Model)**.**

*1.*  *We only observe particle positions ${\overset{★}{\underline{z}}}_{k}^{i,\mathrm{pos}}\in R^{2},i=1,2,\ldots$*
*2.*  *The measurement noise is additive, time-invariant, and Gaussian with covariance $C^{\underline{v}}$, that is, the measurement model is defined by*
  $$\begin{array}{cc} p\;\left({\underline{z}}_{k}^{i,\mathrm{pos}}|{\underline{x}}_{k}^{i,\mathrm{mot}}\right) & =N\left(H{\underline{x}}_{k}^{i,\mathrm{mot}},C^{\underline{v}}\right)\;, \end{array}$$
  *where H extracts the position components.*

The assumptions are typical for vision-based multitarget tracking (see [8]). In particular, we are not given explicit information about measurement-to-track association, track births and deaths, track splits, and clutter.

#### 5.2.2. Actuator Model

The actuator model describes the actuator dynamics given the control inputs. We make the following assumptions:

**Assumption 4** (Actuator Model)**.**

*1.*  *Given ${\underline{a}}_{k-1}$ and ${\underline{u}}_{k-1}$, the actuator state evolves deterministically.*
*2.*  *The actuator dynamics are identical for all actuators $j=1\ldots N_{A}$.*
*3.*  *The actuator states are fully observable or can be perfectly inferred from the actuator model and given controls.*

Thus, the transition model is

$$\begin{array}{c} p\;\left({\underline{a}}_{k}|{\underline{a}}_{k-1},{\underline{u}}_{k-1}\right)=\prod_{j=1}^{N_{A}}\delta \left(a_{k}^{j}-f^{\mathrm{act}}\left(a_{k-1}^{j},u_{k-1}^{j}\right)\right)\;, \end{array}$$

with $f^{\mathrm{act}}\left(\cdot \right)$ as the transition function of the deterministic state-space model that describes a single actuator’s dynamics, $a_{k}^{j}$ as the state of actuator *j* at time step *k*, and $u_{k}^{j}$ as the control input for actuator *j* at time *k*. For our actuator model, we furthermore consider control inputs of the form ${\underline{u}}_{k}={\left[\begin{array}{ccc} u_{k}^{1} & \ldots & u_{k}^{N_{A}} \end{array}\right]}^{⊤}$, where $u_{k}^{j}\in U$ with $U=\left[0,T\right)\cup \left\{\infty \right\}$ describes the activation time of the *j*-th actuator at time step *k*, and *∞* is used to indicate that the actuator is not activated at time step *k* (note that the control input for a single actuator *j* is scalar in the considered case).

The function $f^{\mathrm{act}}\left(\cdot \right)$ describes a cycle of the phases Ready, Activate, Up, Hit, Down, and Reset. Each phase has a fixed duration, $t_{\mathrm{activate}}^{\mathrm{act}},t_{\mathrm{up}}^{\mathrm{act}},t_{\mathrm{hit}}^{\mathrm{act}},t_{\mathrm{down}}^{\mathrm{act}},t_{\mathrm{reset}}^{\mathrm{act}}$, after which the actuator advances to the next phase, except in the Ready phase, which ends only upon receiving a control input. Each phase is associated with a distinct ejection probability, as used in the contact model (Section 5.2.3). The intuition is as follows: Within the Ready phase, the actuator waits without affecting passing particles, i.e., the ejection probability is zero. After activation, the actuator enters the Activate phase and begins moving but does not yet influence particles, for example, because it has not yet reached the region where particles pass by. During Up and Down, the actuator already affects particles, but with a lower ejection probability than in the Hit phase, the preferred ejection phase. In the Reset phase, the actuator returns to Ready; the ejection probability is again zero. This phase sequence captures the periodic actuator dynamics in a computationally efficient form and remains flexible enough to represent different actuator types, including pneumatic nozzles. The periodic structure also motivates Assumption 4 Items 1 and 3: the actuator phase is known whenever the cycle completes, and the actuator returns to rest (i.e., there is no long-term error accumulation). For our impact-based mechanical actuators, we introduce two additional assumptions:

**Assumption 4** (Actuator Model (*Continued*))**.**

*4.*  *Control inputs only affect the actuator if it is in the* Ready *phase, that is, control inputs for actuators in other phases are ignored.*
*5.*  *The sampling interval T, i.e., the time span between two time steps, is smaller than or equal to the duration of one actuator cycle $t_{\mathrm{cycle}}^{\mathrm{act}}=t_{\mathrm{activate}}^{\mathrm{act}}+t_{\mathrm{up}}^{\mathrm{act}}+t_{\mathrm{hit}}^{\mathrm{act}}+t_{\mathrm{down}}^{\mathrm{act}}+t_{\mathrm{reset}}^{\mathrm{act}}$.*

The latter two assumptions imply that each actuator can be activated at most once per time step *k*. Thus, the space spanned by ${\underline{u}}_{k}$ covers all possible activation times within that time step. Because all actuators share the same dynamics, we omit the actuator index *j* in the derivations below for clarity.

**Figure 2:**
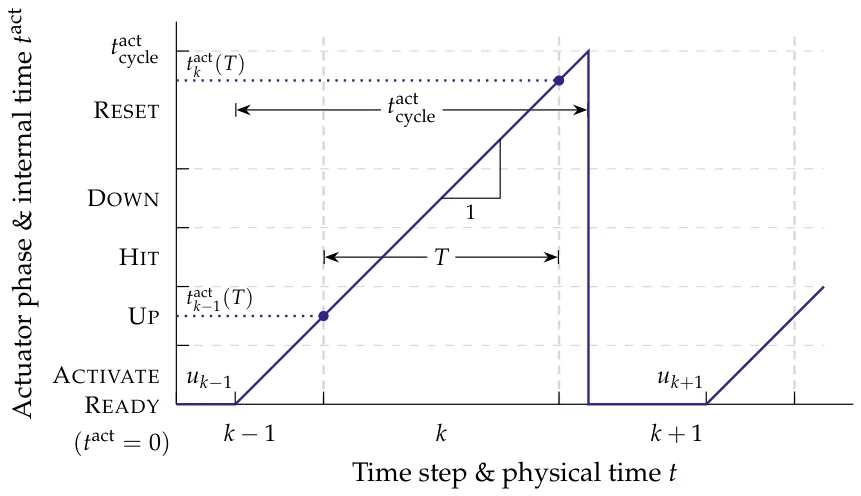
Schematic relation (solid blue line) between physical time *t* and internal actuator time $t^{\mathrm{act}}$ (describing the actuator state) over three consecutive time steps. The horizontal axis represents the time step (outside the plot area) and physical time *t* (inside the plot area), the vertical axis shows the actuator phase (outside the plot area) and internal actuator time $t^{\mathrm{act}}$ (inside the plot area). Arrows indicate time spans, while dotted blue lines link to internal actuator times on the vertical axis for visual clarity. The actuator model follows a cycle of the six strictly consecutive phases: READY (corresponding to $t^{\mathrm{act}}=0$), ACTIVATE, UP, HIT, DOWN, and RESET, each of the latter five is described by its length $t_{\mathrm{activate}}^{\mathrm{act}},t_{\mathrm{up}}^{\mathrm{act}},t_{\mathrm{hit}}^{\mathrm{act}},t_{\mathrm{down}}^{\mathrm{act}}$, and $t_{\mathrm{reset}}^{\mathrm{act}}$, respectively, and corresponding ejection probabilities (for this figure, $t_{\mathrm{activate}}^{\mathrm{act}}=t_{\mathrm{up}}^{\mathrm{act}}=t_{\mathrm{hit}}^{\mathrm{act}}=t_{\mathrm{down}}^{\mathrm{act}}$ and $t_{\mathrm{reset}}^{\mathrm{act}}=2\;\cdot \;t_{\mathrm{activate}}^{\mathrm{act}}$). A segment with slope 1 starting at $t_{k-1}^{\mathrm{act}}\left(T\right)$ corresponds to the case where actuator movement is due to an activation in the previous time step k−1. A segment starting at $u_{k}$ corresponds to the case of an activation in the current time step *k*. Horizontal segments at $t^{\mathrm{act}}=0$ indicate the READY phase.

The state of an actuator is fully described by an internal actuator time $t^{\mathrm{act}}\in \left[0,t_{\mathrm{cycle}}^{\mathrm{act}}\right)$, which describes the time since the start of the current actuator cycle. A trivial mapping $\phi^{\mathrm{act}}:\left[0,t_{\mathrm{cycle}}^{\mathrm{act}}\right)\to$ {Rready, Activate, Up, Hit, Down, Reset} then outputs the actuator phase, taking into account their durations. We model the actuator in continuous time, that is, we use a function

$$\begin{array}{c} {\tilde{f}}^{\mathrm{act}}:\left[0,T\right]\times \left[0,t_{\mathrm{cycle}}^{\mathrm{act}}\right)\times U\to \left[0,t_{\mathrm{cycle}}^{\mathrm{act}}\right)\;,\;\left(t,t_{k-1}^{\mathrm{act}}\left(T\right),u_{k}\right)\mapsto t^{\mathrm{act}} \end{array}$$

that maps an arbitrary time $t\in \left[0,T\right]$, the internal actuator time at the end of the previous time step $t_{k-1}^{\mathrm{act}}\left(T\right)$, and the control input $u_{k}$, to the actuator time $t^{\mathrm{act}}$ at time *t*. To keep the formulas short, *t* is assumed here to be given as delta time with respect to the current time step. The transition function $f^{\mathrm{act}}\left(\cdot \right)$ is then simply ${\tilde{f}}^{\mathrm{act}}\left(T,t_{k-1}^{\mathrm{act}}\left(T\right),u_{k}\right)$, and consequently the (discrete-time) state $a_{k}^{j}$ of actuator *j* is $t_{k-1}^{j,\mathrm{act}}\left(T\right)$. Figure 2 illustrates the relation between physical time *t* and internal actuator time $t^{\mathrm{act}}$ across three consecutive time steps. The exact mathematical form of ${\tilde{f}}^{\mathrm{act}}$ is derived in Appendix A.

In addition to the forward dynamics ${\tilde{f}}^{\mathrm{act}}\left(\cdot \right)$, the contact model will require an inverse actuator model that outputs physical time given an internal actuator time (e.g., the time when a HIT phase begins), that is, we invert ${\tilde{f}}^{\mathrm{act}}\left(\cdot \right)$. This yields a function

$$\begin{array}{c} {\tilde{g}}^{\mathrm{act}}:\left(0,t_{\mathrm{cycle}}^{\mathrm{act}}\right)\times \left[0,t_{\mathrm{cycle}}^{\mathrm{act}}\right)\times U\to \left[0,T\right)\cup \left\{\infty \right\}\;,\;\left(t^{\mathrm{act}},t_{k-1}^{\mathrm{act}}\left(T\right),u_{k}\right)\mapsto t \end{array}$$

that maps the internal actuator time $t^{\mathrm{act}}$, the actuator status at the end of the previous time step $t_{k-1}^{\mathrm{act}}\left(T\right)$, and the control input $u_{k}$ to time *t* (again, for convenience, *t* is assumed to be given as delta time with respect to the current time step). Note that we restricted the domain of ${\tilde{g}}^{\mathrm{act}}\left(\cdot \right)$ to $\left(0,t_{\mathrm{cycle}}^{\mathrm{act}}\right)$. This is because for $t^{\mathrm{act}}=0$, the continuous actuator dynamics are not injective ($t^{\mathrm{act}}=0$ for a wide range of *t*, $t_{k-1}^{\mathrm{act}}\left(T\right)$, and $u_{k}$), while for all $t^{\mathrm{act}}\in \left(0,t_{\mathrm{cycle}}^{\mathrm{act}}\right)$ the mapping is bijective. Again, we refer to Appendix A for the mathematical form of ${\tilde{g}}^{\mathrm{act}}$.

#### 5.2.3. Contact Model

The contact model describes $P\;\left({\mathrm{ej}}_{k}^{i}=1|{\underline{x}}_{k}^{i,\mathrm{mot}},{\underline{a}}_{k},{\underline{u}}_{k}\right)$, where the dependence on the actuators and the control input is deterministic. In the following, we omit the time indices *k* since the contact model is static and therefore identical for all time steps.

A particle can be newly ejected at an actuator only if it has not already been ejected upstream. Unfolding the corresponding recurrence over the actuator sequence yields

$$\begin{array}{c} P\;\left({\mathrm{ej}}^{i}=1|{\underline{x}}^{i,\mathrm{mot}},\underline{a},\underline{u}\right)=1-\prod_{j=1}^{N_{A}}\left(1-P\;\left({\mathrm{ej}}^{i,j}=1|{\underline{x}}^{i,\mathrm{mot}},a^{j},u^{j}\right)\right)\;, \end{array}$$

i.e., the ejection probability equals one minus the probability of surviving every actuator. In particular, it is independent of the actuator order, since each factor $P\;\left({\mathrm{ej}}^{i,j}=1|a^{j},u^{j}\right)$ depends only on actuator *j*. The distribution $P\;\left({\mathrm{ej}}^{i,j}=1|{\underline{x}}^{i,\mathrm{mot}},a^{j},u^{j}\right)$ is modeled as

$$\begin{array}{c} P\;\left({\mathrm{ej}}^{i,j}=1|{\underline{x}}^{i,\mathrm{mot}},a^{j},u^{j}\right)=\sum_{m\in \Omega_{{\mathrm{in}}^{i,j}}}P\;\left({\mathrm{ej}}^{i,j}=1|in^{i,j}=m\right)P\;\left({\mathrm{in}}^{i,j}=m|{\underline{x}}^{i,\mathrm{mot}},a^{j},u^{j}\right)\;, \end{array}$$

where we introduce the auxiliary variable ${\mathrm{in}}^{i,j}\in \Omega_{{\mathrm{in}}^{i,j}}$ and $\Omega_{{\mathrm{in}}^{i,j}}=$ {Hit, Up, Down, else} with ${\mathrm{in}}^{i,j}=m≜$ “particle *i* in the influence range of actuator *j* at phase *m*” for all $m\in \Omega_{{\mathrm{in}}^{i,j}}$. For the first term, we use

$$\begin{array}{c} P\;\left({\mathrm{ej}}^{i,j}=1|in^{i,j}=m\right)=p_{\mathrm{eject}},m\;,\;\forall m\in \Omega_{{\mathrm{in}}^{i,j}}\;, \end{array}$$

with $p_{\mathrm{eject},\mathrm{else}}=0$ by definition. Here, $p_{\mathrm{eject},\mathrm{HIT}}$, $p_{\mathrm{eject},\mathrm{UP}}$, and $p_{\mathrm{eject},\mathrm{DOWN}}$ describe the probabilities of ejection in the Hit, Up, and Down phases, respectively.

The conditional distribution of ${\mathrm{in}}^{i,j}$ given $\tau^{i,j}$, the arrival time of particle *i* at actuator *j*, and $y^{i}\left(\tau^{i,j}\right)$, i.e., the y-position of particle *i* when it arrives at actuator *j*, is assumed to be

$$\begin{array}{c} P\;\left({\mathrm{in}}^{i,j}=m|\tau^{i,j},y^{i}\left(\tau^{i,j}\right),a^{j},u^{j}\right)=\left\{\begin{array}{cc} 1, & {\tilde{g}}^{\mathrm{act}}\left(t_{m,\mathrm{begin}}^{\mathrm{act}},a^{j},u^{j}\right)\leq \tau^{i,j}\leq {\tilde{g}}^{\mathrm{act}}\left(t_{m,\mathrm{end}}^{\mathrm{act}},a^{j},u^{j}\right)\\ \; & \;\wedge \;y^{\mathrm{act},j}-w^{\mathrm{act}}\;/\;2<y^{i}\left(\tau^{i,j}\right)<y^{\mathrm{act},j}+w^{\mathrm{act}}\;/\;2\;,\\ 0, & \mathrm{else}\;. \end{array}\right. \end{array}$$

Here, ${\tilde{g}}^{\mathrm{act}}\left(\cdot \right)$ is as defined in Section 5.2.2 and returns the start and end time of the actuator phase *m*, that is, the physical time when an actuator has internal time $t_{m,\mathrm{begin}}^{\mathrm{act}}$ and $t_{m,\mathrm{end}}^{\mathrm{act}}$, respectively. For example, $t_{\mathrm{HIT},\mathrm{begin}}^{\mathrm{act}}=t_{\mathrm{activate}}^{\mathrm{act}}+t_{\mathrm{up}}^{\mathrm{act}}$ and $t_{\mathrm{HIT},\mathrm{end}}^{\mathrm{act}}=t_{\mathrm{activate}}^{\mathrm{act}}+t_{\mathrm{up}}^{\mathrm{act}}+t_{\mathrm{hit}}^{\mathrm{act}}$, and we define $t_{\mathrm{else},\mathrm{begin}}^{\mathrm{act}}=t_{\mathrm{else},\mathrm{end}}^{\mathrm{act}}$. Furthermore, $y^{\mathrm{act},j}$ denotes the y-position of actuator *j*, and $w^{\mathrm{act}}$ denotes the width of actuators (in y-direction). Therefore, the probability is only nonzero if the particle arrives in a rectangle with length equal to the duration of the current phase and width equal to the actuator width. We use the Gauß–Taylor approximations to obtain the distributions $p\;\left(\tau^{i,j}\right)$ and $p\;\left(y^{i}\left(\tau^{i,j}\right)\right)$ from $p\;\left({\underline{x}}^{i,\mathrm{mot}}\right)$ and the actuator index *j* (details in [9,28]). Since the Gauß–Taylor approximations are deterministic linear transformations of the state ${\underline{x}}^{i,\mathrm{mot}}$, the joint density $p\;\left(\tau^{i,j},y^{i}\left(\tau^{i,j}\right)\right)$ is again Gaussian with known mean and covariance.

### 5.3. Assumed Density Filtering-Inspired Prediction

Given the stochastic system model from Section 5.2, prediction within the OLF control horizon requires a recursive representation of the joint distribution $p\;\left({\underline{x}}_{k}^{i,\mathrm{mot}},ex_{k}^{i}\right)$ of the motion and existence state of particle *i*. (The actuator state ${\underline{a}}_{k}$ is omitted from this joint distribution because it evolves deterministically.) In the following, we omit the particle index *i* for brevity since particles are assumed to be i.i.d. by Assumption 2 Item 1. We likewise suppress the deterministic conditioning on ${\underline{a}}_{k-1}$ and ${\underline{u}}_{k-1}$ in the existence transition.

Let $p^{\left(s\right)}\left({\underline{x}}_{k}^{\mathrm{mot}}\right)=p\;\left({\underline{x}}_{k}^{\mathrm{mot}},ex_{k}=s\right)$ denote the unnormalized continuous joint sub-density in existence mode ${\mathrm{ex}}_{k}=s$, s∈{0,1}. By the law of total probability, the exact recursion is

$$\begin{array}{c} p^{\left(r\right)}\left({\underline{x}}_{k}^{\mathrm{mot}}\right)=\int_{R^{n_{\underline{x}}}}p\;\left({\underline{x}}_{k}^{\mathrm{mot}}|{\underline{x}}_{k-1}^{\mathrm{mot}}\right)\sum_{s\in \{0,1\}}P\;\left({\mathrm{ex}}_{k}=r|{\underline{x}}_{k-1}^{\mathrm{mot}},ex_{k-1}=s\right)p^{\left(s\right)}\left({\underline{x}}_{k-1}^{\mathrm{mot}}\right)\;d{\underline{x}}_{k-1}^{\mathrm{mot}} \end{array}$$

for r∈{0,1}. The transition probability $P\;\left({\mathrm{ex}}_{k}=r|{\underline{x}}_{k-1}^{\mathrm{mot}},ex_{k-1}=s\right)$ is induced by the contact model and is piecewise constant with values ≥0 over regions of the motion state. It therefore weights and truncates the prior sub-density $p^{\left(s\right)}\left({\underline{x}}_{k-1}^{\mathrm{mot}}\right)$.

Let us assume for a moment that $P\;\left({\mathrm{ex}}_{k}=0|{\underline{x}}_{k-1}^{\mathrm{mot}},ex_{k-1}=s\right)$ is a single step function, i.e., $P\;\left({\mathrm{ex}}_{k}=0|{\underline{x}}_{k-1}^{\mathrm{mot}},ex_{k-1}=s\right)=1_{S_{k-1}^{\left(s\right)}}\left(\underline{\psi }\left({\underline{x}}_{k-1}^{\mathrm{mot}}\right)\right)$, where $\underline{\psi }:R^{n_{\underline{x}}}\to R^{n_{\underline{\xi }}}$ is an arbitrary transformation and $S_{k-1}^{\left(s\right)}\subseteq R^{n_{\underline{\xi }}}$, s∈{0,1}, are arbitrary Borel sets. Then, even if both prior sub-densities $p^{\left(s\right)}\left({\underline{x}}_{k-1}^{\mathrm{mot}}\right)$ are Gaussian, $p^{\left(r\right)}\left({\underline{x}}_{k}^{\mathrm{mot}}\right)$ is a mixture of two non-Gaussian components (because of the truncation by the step function). Each further recursion therefore doubles the number of mixture components and is further complicated by non-Gaussianity. In the simplest setting—when considering only one component per mode, linear maps, and one-sided boundaries—the resulting densities belong to the unified skew-normal family [49], whose parameter dimension grows with every recursion so that evaluation quickly becomes intractable. Consequently, no computationally tractable exact recursion is available for this class of hybrid-state systems.

We therefore adopt an assumed density filtering (ADF)-inspired prediction. At each step, the unnormalized sub-densities are approximated by scaled Gaussians

$$\begin{array}{c} p^{\left(s\right)}\left({\underline{x}}_{k-1}^{\mathrm{mot}}\right)\approx {\tilde{\pi }}_{k-1}^{\left(s\right)}N\left({\underline{x}}_{k-1}^{\mathrm{mot}};{\underline{\mu }}_{k-1}^{\left(s\right)},P_{k-1}^{\left(s\right)}\right)\;. \end{array}$$

The approximate recursion proceeds in three phases:

1. Weighting and truncation. Directly after applying the existence transition, define the non-Gaussian intermediate density $q^{\left(r\right)}\left({\underline{x}}_{k-1}^{\mathrm{mot}}\right)=p\;\left({\underline{x}}_{k-1}^{\mathrm{mot}},ex_{k}=r\right)$ by

$$\begin{array}{c} q^{\left(r\right)}\left({\underline{x}}_{k-1}^{\mathrm{mot}}\right)=\sum_{s\in \{0,1\}}P\;\left({\mathrm{ex}}_{k}=r|{\underline{x}}_{k-1}^{\mathrm{mot}},ex_{k-1}=s\right)p^{\left(s\right)}\left({\underline{x}}_{k-1}^{\mathrm{mot}}\right)\;. \end{array}$$

2. Moment matching. The weighted and truncated density $q^{\left(r\right)}$ is replaced by a single scaled Gaussian through matching of probability mass ${\tilde{\pi }}_{k}^{\left(r\right)}$, mean ${\tilde{\underline{\mu }}}_{k-1}^{\left(r\right)}$, and covariance ${\tilde{P}}_{k-1}^{\left(r\right)}$. Closed-form expressions exist for special cases such as linear maps $\underline{\psi }\left(\cdot \right)$ and rectangular regions $S_{k-1}^{\left(s\right)}$ (see Appendix B). Appendix C shows that, under mild geometric assumptions that hold to a very good approximation for the staggered grid, the contact model belongs to this class and derives the corresponding moments.

3. Continuous prediction. With ${\tilde{\underline{\mu }}}_{k-1}^{\left(r\right)}$ and ${\tilde{P}}_{k-1}^{\left(r\right)}$, the linear motion model yields

$$\begin{array}{c} {\underline{\mu }}_{k}^{\left(r\right)}=A{\tilde{\underline{\mu }}}_{k-1}^{\left(r\right)}\;,\;P_{k}^{\left(r\right)}=A{\tilde{P}}_{k-1}^{\left(r\right)}A^{⊤}+C^{\underline{w}}\;. \end{array}$$

This, for the next time step *k*, re-establishes the closed scaled-Gaussian recursion

$$\begin{array}{c} p^{\left(r\right)}\left({\underline{x}}_{k}^{\mathrm{mot}}\right)\approx {\tilde{\pi }}_{k}^{\left(r\right)}N\left({\underline{x}}_{k}^{\mathrm{mot}};{\underline{\mu }}_{k}^{\left(r\right)},P_{k}^{\left(r\right)}\right)\;. \end{array}$$

### 5.4. Cost Evaluation

We consider terminal costs only ($g_{k,n}\left(\cdot \right)=0$ for n<N). The terminal penalty counts falsely sorted accept and reject particles according to

$$\begin{array}{cc} g_{k,N}\left({\underline{x}}_{k,N}\right)\; & =c_{\mathrm{acc}}\sum_{i=1}^{N_{P}}1_{\left\{c^{i}=\mathrm{acc}\wedge {\mathrm{ex}}_{k,N}^{i}=0\right\}}+c_{\mathrm{rej}}\sum_{i=1}^{N_{P}}1_{\left\{c^{i}=\mathrm{rej}\wedge {\mathrm{ex}}_{k,N}^{i}=1\right\}}\;, \end{array}$$

where $1_{\left\{\cdot \right\}}$ is the indicator function and $c_{\mathrm{acc}},c_{\mathrm{rej}}\geq 0$ are weights, typically with $c_{\mathrm{acc}}+c_{\mathrm{rej}}=1$. Under OLF and with $g_{k,n}\left(\cdot \right)=0$ for n<N, Equation (3) then reduces to

$$\begin{array}{c} J_{k}\left({\tilde{I}}_{k,N}\right)=\sum_{i=1}^{N_{P}}c_{\mathrm{acc}}1_{\left\{c^{i}=\mathrm{acc}\right\}}P\;\left({\mathrm{ex}}_{k,N}^{i}=0|{\tilde{I}}_{k,N}\right)+\sum_{i=1}^{N_{P}}c_{\mathrm{rej}}1_{\left\{c^{i}=\mathrm{rej}\right\}}P\;\left({\mathrm{ex}}_{k,N}^{i}=1|{\tilde{I}}_{k,N}\right)\;, \end{array}$$

where we explicitly indicate the dependence on ${\tilde{I}}_{k,N}$, and hence on the considered control sequence, and exploit that the expectation over ${\mathrm{ex}}_{k,N}^{i}$ can be evaluated in closed form. Here, $P\;\left({\mathrm{ex}}_{k,N}^{i}=1|{\tilde{I}}_{k,N}\right)=P\;\left({\mathrm{ex}}_{k,N}^{i}=1|p\;\left({\underline{x}}_{0}\right)p\;\left({\underline{x}}_{0}\right),{\underline{u}}_{0:k+N-1},{\overset{★}{\underline{z}}}_{0:k}\right)$ is the predicted existence probability at stage *N* given measurements up to time step *k*. It is obtained from ${\tilde{\pi }}_{k,N}^{\left(1\right)}$ by multitarget tracking on position measurements up to *k*, followed by recursive prediction to stage *N* (see Section 5.3). Intuitively, $J_{k}\left({\tilde{I}}_{k,N}\right)$ sums the predicted probabilities that accept or reject particles are in the wrong existence state at stage *N*.

Appendix D first numerically verifies the underlying ADF Gaussians and the corresponding open-loop costs against fine-*T* Monte Carlo under realistic tracking uncertainty: the ADF typically matches the Monte Carlo reference distributions well, with residual mismatches mainly right after contact or under high uncertainty, while cost deviations remain about two orders of magnitude smaller than the terminal penalties. Mismatches matter little for sequence selection for two reasons: (i) process noise restores near-Gaussian shapes within a few prediction steps, so at terminal stage *N* the ADF typically approximates the Monte Carlo reference distributions closely, and (ii) the stochastic cost-to-go down-weights poorly observed tracks—unlike a deterministic MPC cost on a single nominal trajectory.

The preceding numerical verification uses identical model settings and state information for the ADF and the Monte Carlo reference. The complementary sensitivity analysis in Appendix D.3 instead evaluates selected cases in which the Monte Carlo truth model differs from the model used by the ADF. The results indicate comparatively small effects for the tested perturbations of phase durations and ejection probabilities, whereas the process-noise covariance and the particle-state covariance supplied by the tracker are more sensitive parameters.

### 5.5. Trajectory Generation

Trajectory generation builds candidate control sequences in $C_{k}$ (Section 5.1.1) that, preferably, have low expected cost. Each candidate consists of particle–actuator assignments, with at most one actuator assigned to each particle; the full set of ${\left(N_{A}+1\right)}^{N_{P}^{\mathrm{rej}}}$ sequences can be viewed as a decision tree whose nodes are partial assignments (including “no actuator”) and whose root-to-leaf paths are complete sequences. Exhaustive enumeration is infeasible, so we combine:

- Pruning to drop infeasible branches (e.g., when a particle cannot be reached by an actuator);
- Rollout to limit search depth by greedily completing partial control sequences (partial paths in the tree).

**Figure 3:**
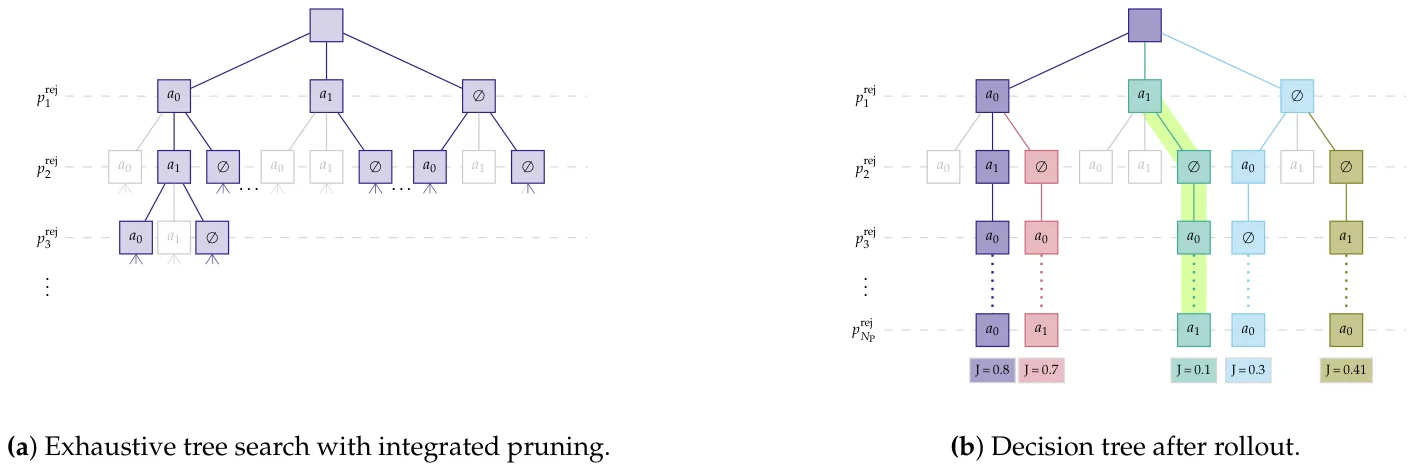
Decision trees after exhaustive search with pruning (**a**) and after rollout (**b**) (example with two actuators). Nodes are assignments of reject particles $p_{1}^{\mathrm{rej}},\ldots$ to an actuator or ∅; gray branches are pruned. Complete candidates are root-to-leaf paths; in (**b**), colored paths denote rollout completions, with the yellow highlight marking the minimum-cost sequence applied to the system.

Figure 3 illustrates the two phases: exhaustive search over the first $N_{R}$ reject particles with integrated pruning (Figure 3a), then rollout completion to assign the remaining particles to actuators (Figure 3b).

Because tree search and rollout treat particles differently, the assignment of particles to each stage matters. Ideally, hard-to-separate particles would be searched exhaustively, and particles in locally low occupation density would be assigned to the greedy heuristic. Identifying such particles is itself combinatorial, so we use a simple heuristic: reject particles are sorted by increasing distance to the nearest accept particle, assuming that these particles are harder to separate, and the first $N_{R}$ are assigned to the exhaustive tree search.

#### 5.5.1. Phase 1: Exhaustive Tree Search with Integrated Pruning

An assignment of particle *i* to actuator *j* is valid only if the following conditions hold:

1. Be unaffected. A particle’s trajectory must not be affected by upstream actuators. That is, for every actuator *l* that particle *i* passes before reaching actuator *j*, it must hold that ${\tilde{\phi }}^{\mathrm{act}}\left({\hat{\tau }}^{i,l},t_{k-1}^{l,\mathrm{act}}\left(T\right),{\tilde{u}}_{k,0:N-1}^{l}\right)\notin$ {Hit, Up, Down}, where ${\tilde{\phi }}^{\mathrm{act}}\left(\cdot \right)$ denotes the actuator phase at the expected passage time ${\hat{\tau }}^{i,l}$, given the actuator state $t_{k-1}^{l,\mathrm{act}}\left(T\right)$ at time step *k* and the (already scheduled) control sequence ${\tilde{u}}_{k,0:N-1}^{l}$ along the current tree path. Here, the mapping ${\tilde{\phi }}^{\mathrm{act}}$ is trivially given by recursively calling ${\tilde{f}}^{\mathrm{act}}\left(\cdot \right)$ until ${\hat{\tau }}^{i,l}$ is reached and subsequently evaluating $\phi^{\mathrm{act}}\left(\;\cdot \;\right)$ on the result.

2. Be ready. Actuator *j* must be in the READY phase at activation. This requires that ${\tilde{\phi }}^{\mathrm{act}}\left({\hat{\tau }}^{i,j}-t_{\mathrm{lead}}^{\mathrm{act}},t_{k-1}^{j,\mathrm{act}}\left(T\right),{\tilde{u}}_{k,0:N-1}^{j}\right)=$ Ready, with lead time $t_{\mathrm{lead}}^{\mathrm{act}}=t_{\mathrm{activate}}^{\mathrm{act}}+t_{\mathrm{up}}^{\mathrm{act}}+t_{\mathrm{hit}}^{\mathrm{act}}\;/\;2$ to reach the center of HIT at predicted arrival ${\hat{\tau }}^{i,j}$ (Assumption 1 Item 2).

3. Be free. Before the next scheduled activation on the same path, actuator *j* must finish its cycle, that is, ${\tilde{\phi }}^{\mathrm{act}}\left({\hat{\tau }}^{i,j}+t_{\mathrm{cycle}}^{\mathrm{act}}-t_{\mathrm{lead}}^{\mathrm{act}},t_{k-1}^{j,\mathrm{act}}\left(T\right),{\tilde{u}}_{k,0:N-1}^{j}\right)=$ Ready.

Although these conditions greatly reduce the search space, it can still remain too large for online evaluation. Moreover, the number of surviving candidate solutions—and thus the runtime—is stochastic and not known a priori. We therefore cap the number of valid child branches per node at $N_{B}$, which, however, makes the result depend on particle and actuator order. Again, we use the particle order from above and process actuators row by row (upstream first).

#### 5.5.2. Phase 2: Heuristic Rollouts

Remaining particles are assigned with the greedy FAF heuristic: each reject particle is mapped to the first actuator it is predicted to pass that can still be activated in time (based on the same expected arrival times ${\hat{\tau }}^{i,j}$ as in the tree search). After each assignment, actuator states are updated before processing the next particle. If only one actuator is feasible, it is chosen automatically. By default, particles are processed in order of appearance in the camera FOV. FAF is cheap but myopic: it does not coordinate globally, account for accept particles, or compare competing assignments.

The sequences obtained by tree search with FAF completion form a candidate set ${\left\{{\underline{u}}_{k,0:N-1}^{\left(i\right)}\right\}}_{i=1,2,\ldots }$ from which the minimum-cost sequence is selected (Algorithm 1). Under the order-dependent branch cap $N_{B}$, the pure FAF base sequence need not appear in the candidate set, so the usual rollout guarantee that the selected sequence is not worse than the base heuristic [22,23,24] does not hold automatically. As an optional safeguard, the standalone FAF sequence ${\underline{u}}_{k,0:N-1}^{\mathrm{FAF}}$ can be appended to ${\left\{{\underline{u}}_{k,0:N-1}^{\left(i\right)}\right\}}_{i=1,2,\ldots }$ if it is missing, which restores $J_{k}\left({\underline{u}}_{k,0:N-1}^{*}\right)\leq J_{k}\left({\underline{u}}_{k,0:N-1}^{\mathrm{FAF}}\right)$ under the OLF cost-to-go used for ranking. An ablation (Appendix E) shows that this option has negligible effect on closed-loop sorting metrics, so all reported simulation and laboratory runs omit it.

### 5.6. Algorithm and Implementation Details

Algorithm 1 summarizes one receding-horizon step of MTCR. At time step *k*, predictive tracking updates particle states from position measurements and calculates expected arrival times at all actuators; actuator states are propagated from ${\underline{u}}_{k-1}$ via the actuator model (Section 5.2.2). A breadth-first search builds candidates (Section 5.5), their OLF cost (Section 5.4) is evaluated, and the minimum-cost sequence is applied. Our implementation allows the controller call rate to differ from the discrete-time step used for internal prediction and cost evaluation, provided the latter remains at most $t_{\mathrm{cycle}}^{\mathrm{act}}$.
**Algorithm 1** MTCR-step at time step *k*
**Input:** Particle position measurements at time step *k*, applied controls ${\underline{u}}_{k-1}$, optional flag to retain the FAF base sequence in C (default: off)**Output:** Control sequence ${\underline{u}}_{k,0:N-1}^{*}$ with minimum cost//Update particle and actuator states1:Estimate particle states and arrival times from measurements via predictive tracking2:Propagate actuator states to step *k* from ${\underline{u}}_{k-1}$ via the actuator model (Section 5.2.2)//Breadth-first search for generating control sequences3:Sort reject particles (e.g., ascending by distance to nearest accept particle)4:Initialize queue Q←{(0,∅)} and candidate list C←∅5:**while**Q not empty **do**6:    Dequeue node (i,j) from Q7:    **if** $i>N_{R}-1$ **or** all particles were handled **then**8:        Trace back ancestors of (i,j) to extract (partial) control sequence, complete with FAF rollout $\to {\underline{u}}_{k,0:N-1}$; append to C9:        **continue**10:    **end if**11:    **for** each actuator $j^{\prime }\in \left\{1,\ldots ,N_{A}\right\}\cup \left\{\emptyset \right\}$ **do**12:        **if** (i,j) already has $N_{B}$ children **then**13:           **break**14:        **end if**15:        **if** all ejection conditions from Section 5.5.1 are satisfied for reject particle i+1 (in the sorted order) at actuator $j^{\prime }$ **then**16:           Enqueue child node $\left(i+1,j^{\prime }\right)$ into Q, keep track of the parent node (i,j)17:        **end if**18:    **end for**19:**end while**20:**if** retain FAF base sequence **then**21:    Compute standalone FAF sequence ${\underline{u}}_{k,0:N-1}^{\mathrm{FAF}}$; append to C if not already present22:**end if**//Cost evaluation and selection23:Select ${\underline{u}}_{k,0:N-1}^{*}\leftarrow {arg min}_{{\underline{u}}_{k,0:N-1}\in C}J_{k}\left({\underline{u}}_{k,0:N-1}\right)$ with $J_{k}\left(\cdot \right)$ as in Section 5.4

## 6. Prototype Grid Sorter ISAS-TableSort

**Figure 4:**
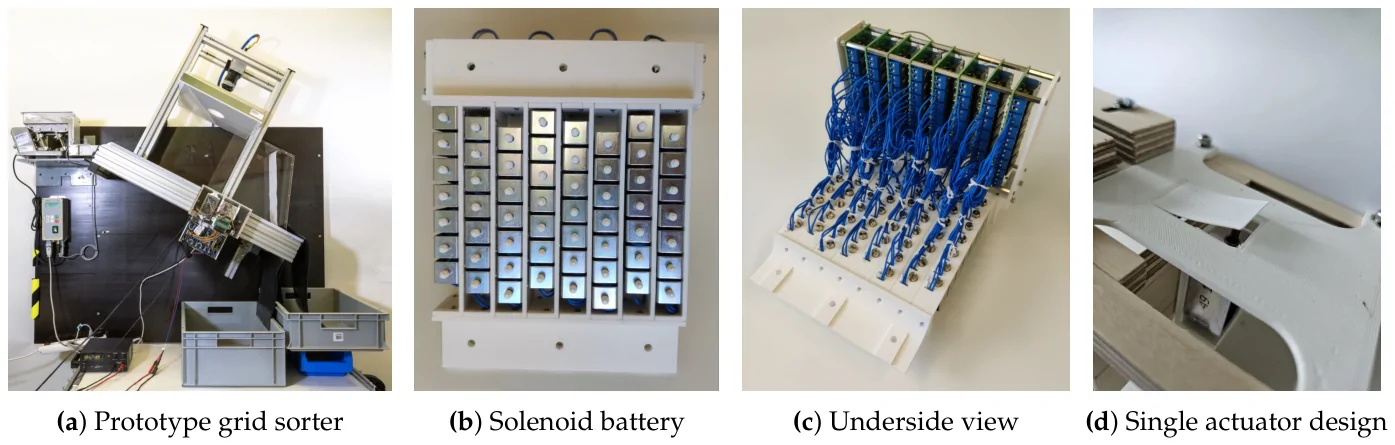
Prototype grid sorter and actuator assembly. (**a**) Laboratory grid sorter. Particles are fed by a vibration feeder, slide down a chute while being tracked in the FOV of an area-scan camera, may be ejected upward by the actuators, and are finally collected in the right (ejected) or left (kept) bin. (**b**) Solenoid battery (dispatch unit with the chute surface removed, including the embedded actuators), and (**c**) dispatch unit from below. (**d**) Design of an individual actuator: particles move from left to right along a flexible flap rigidly connected to the chute surface and deflected by the solenoid; in the rest position, the plunger remains below the flap without contact.

The ISAS-TableSort (see Figure 4) is a laboratory chute sorter with a two-dimensional grid of mechanical actuators on a chute of width 123 mm and inclination 30 °. It comprises 56 actuators arranged in 8 rows of 7, which eject particles upward by momentum exchange. Ejected particles are separated from those remaining in the chute by a separating plate (see also Figure 1a) and guided into different collection bins. Particle detection is performed by a Bayer RGB area-scan camera of type BONITO PRO X-1250C with flat-dome illumination. Its FOV covers a region of about 210 mm directly upstream of the actuator array. Images are acquired at 3008 × 1952 px and 100 fps. Typical particles traverse the FOV at an average speed of about 1.1 m/s.

The dispatch unit, shown in Figure 4b,c, is the core component of the prototype. It measures 120 mm in width and 160 mm in length. Each actuator drives a flexible flap of size 18 × 9 mm embedded in the chute surface, with 20 mm spacing between neighboring rows; laterally, the actuators within each row are spaced 16 mm apart. The actuators are offset in transport direction to increase spatial resolution. The resulting geometry was obtained offline by optimizing actuator extents and inter-actuator and inter-row spacing under geometric constraints. For this, a genetic algorithm scored candidate layouts using a simulation model of the sorting system (see Section 7.1) under FAF control, maximizing separation accuracy. A demonstration video of the prototype in operation is available at https://www.youtube.com/watch?v=Qb6dKNTsn6M (accessed on 19 August 2026); the mass flow in the video differs from the laboratory experiments reported below.

### 6.1. Actuator Design

The ISAS-TableSort uses linear motors, specifically the pushing solenoid Red Magnetics ITS-LS 1614-D, to drive the mechanical actuators. Solenoids operate according to the reluctance principle: when the coil is energized, the magnetic field moves the iron armature, while spring force returns it after de-energization. This simple actuation principle enables a compact and cost-effective design. The chosen solenoid has width 16 mm, height 14 mm, and stator depth 32 mm, with a maximum force of about 7 N. Its unit price is approximately 11 € (as of 2026).

**Figure 5:**
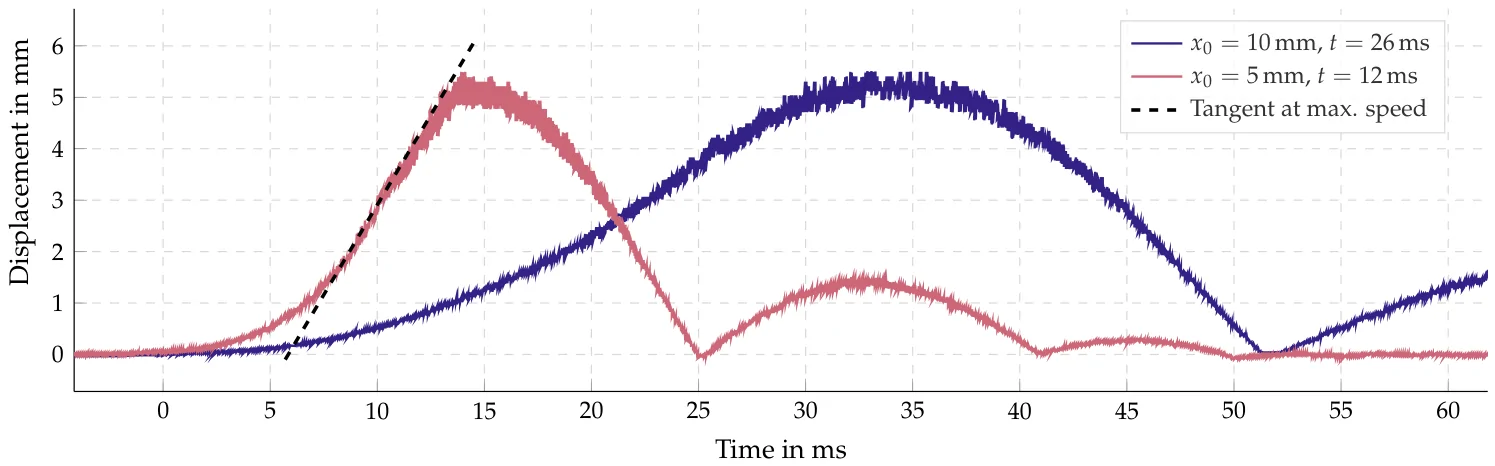
Deflection time course (unloaded) of the solenoid RED MAGNETICS ITS-LS 1614-D, with activation duration *t* chosen such that a 5 mm displacement is achieved. In blue, the deflection duration for a solenoid with a maximum stroke of 10 mm ($x_{0}=10\mathrm{mm}$ and activation duration t=26ms) is shown, and in red for a solenoid with a maximum stroke of 5 mm. The dashed black line describes the tangent at the maximum solenoid velocity.

The linear motor is driven by a rectangular voltage pulse of 30 V for 12 ms, resulting in a design energy of about 0.15 J per activation; measured run-level solenoid energies are reported in Section 7.6. Mechanical stops limit the solenoid stroke such that, in the de-energized rest position, the armature already lies 5 mm inside the coil. This shortens deflection time and increases achievable velocity (see Figure 5). One actuator cycle lasts about 25 ms, allowing actuation frequencies up to 40 Hz. By conservation of momentum, the intended particle contact occurs at maximum actuator velocity, about 8.5 ms after activation and about 2 ms after deflection; this is marked by the black line in Figure 5. The corresponding velocity is about 0.7 m/s. For example, after an impact of the 8 g armature and a 1 g particle, the particle normal velocity ranges from 0.62 m/s to 1.24 m/s, depending on the coefficient of restitution.

To realize impact at this design contact time, the actuators are arranged such that, in the de-energized state, the solenoid armature lies 2 mm below the flexible flap connected to the chute surface (see Figure 4d). This separates the actuator acceleration and return phases from the actual impact and reduces actuator engagement time in the particle flow. Consistently, actuator activation is scheduled about 8.5 ms before the desired impact time. The flap itself is an L-shaped flexible plastic profile rigidly connected to the dispatch-unit cover plate on its short side (see Figure 4d), ensuring dust-tight closure along the direction of motion. When inactive, it seals flush with the chute surface.

### 6.2. Computational Hardware, Software, and Pipelining

The area-scan camera communicates via CoaXPress with a Basler microEnable 5 marathon ACX-QP frame grabber in a PC. Images are then transferred to an Nvidia RTX 3080 GPU (10,240MB VRAM) for particle detection. Further processing and control-input computation run on an Intel®Core™I9-13900KS CPU (8 performance cores at 3.2 GHz and 16 efficiency cores at 2.4 GHz). The PC communicates with an Arduino Uno via Ethernet to control the mechanical actuators.

The controller and all major software components are implemented in Python and accelerated with Numba to simplify integration of prototype components. To achieve real-time performance, the software supports pipelined execution of the main processing steps and, in particular, parallel execution of multiple controller instances that alternate across time steps. In this scheme, the internal actuator model is shared, and older control inputs targeting a specific particle are replaced by more recent ones whenever the updated activation times remain feasible. On the prototype, this pipelined stack is operated at the camera frame rate of 100 fps. A controller is considered real-time capable if it can sustain successful sorting operations at this rate when parallel instances are accounted for; as a rule of thumb, a single call should stay roughly within the corresponding 10 ms frame budget.

## 7. Evaluation

We compare MTCR with FAF in simulation and on the laboratory prototype on tasks with a high fraction of particles to be ejected; in the experiments, we additionally include a high-precision pneumatic reference system. FAF uses the predicted arrival times from predictive tracking, analogously to MTCR. We take accept particles as the positive class and reject particles as the negative class, and report separation accuracy using true negative rate (TNR) and true positive rate (TPR),

$$\begin{array}{c} \mathrm{TNR}=\frac{\mathrm{TN}}{N}\;,\;\mathrm{TPR}=\frac{\mathrm{TP}}{P}\;, \end{array}$$

where TN and TP denote the number of true negative and true positive particles, *N* is the total number of reject particles, and *P* is the total number of accept particles. Both metrics lie in [0,1]; higher values are preferred. TNR measures the rejection of undesired particles, and TPR measures the avoidance of bycatch.

### 7.1. Simulation Model and Settings

The simulation model (see Figure 1b for a visualization) is a slightly adapted, fully discrete-time version of the model in Section 5.2. The actuator model matches Section 5.2.2. Particles follow the model of Section 5.2.1 with CV motion. They spawn in a region upstream of the grid; lateral positions are uniform, and the number of spawns per step follows a clipped normal distribution. Instead of running predictive tracking on sampled measurements, the controller receives ground-truth particle states and an artificial noise covariance, and internally predicts the expected arrival times (resulting in the same arrival times for both MTCR and FAF). Results are therefore optimistic and emphasize combinatorial optimization performance over exact timing.

The simulation contact model differs from the controller contact model (Section 5.2.3) by being deterministic. Contact regions are defined around each actuator’s x-position; a particle is in contact if it lies within distance $d_{\mathrm{contact}}$ and within the actuator width in y-direction at time step *k*. Ejection occurs when the particle is in contact and the actuator is in the Hit phase. Particles that come close to ejection without entering Hit, e.g., during Up or Down, are labeled DISRUPTED and excluded from TN and TP, but reduce TNR or TPR through the denominators *N* and *P*. Particles crossing a wider impact region are also marked Disrupted if the actuator is in Hit, Up, or Down and no successful ejection by the same actuator follows in the next steps.

The simulated geometry matches the experimental prototype with 56 actuators in 8 rows. We simulate 100,000 particles with equal accept and reject fractions. Two mass-flow scenarios are considered, both with a mean particle x-velocity of 1.0 m/s, and parameterized by the mean μ and standard deviation σ of the spawn distribution:

- Medium mass flow: μ≈0.32, σ=0.3, corresponding to roughly 20 particles in the grid (visible and ejected), or about 100/s particles.
- High mass flow: μ=0.64, σ=0.6, corresponding to roughly 40 particles in the grid, or about 200/s particles (see Figure 1b).

MTCR uses a sampling interval of $T=t_{\mathrm{cycle}}^{\mathrm{act}}$, i.e., the maximum allowable time between steps, and horizon N=10, chosen such that particles typically leave the grid within the horizon. The internal motion model is CV. Tree search considers $N_{R}=N_{B}\in \left\{2,4\right\}$, i.e., up to two or four reject particles with at most the same number of branches per node. The controller was evaluated at cost weights $c=c_{\mathrm{rej}}=1-c_{\mathrm{acc}}\in \left\{0.5,0.8\right\}$ in both mass-flow scenarios. Controller parameters were fixed from preliminary experiments prior to the reported evaluation runs. Reported MTCR runs do not force the standalone FAF sequence into the candidate set (optional retention off; see Appendix E). The main parameters used to reproduce the simulation results, including the particle, actuator, and contact model, the controller settings, and the simulator configuration are also tabulated in Table A3.

### 7.2. Simulation Results

Table 1 summarizes sorting metrics and per-step controller runtime (median, third quartile, and maximum latency) (evaluated on a single core of an Intel®Core™I9-9900K CPU at 3.60 GHz). Along with TNR and TPR, we report the DISRUPTED rates $\mathrm{DNR}=D_{N}/N$ and $\mathrm{DPR}=D_{P}/P$, where $D_{N}$ and $D_{P}$ are the numbers of disrupted reject and accept particles, respectively. All MTCR variants outperform FAF in TNR and TPR at both mass-flow levels and achieve lower DNR/DPR. Importantly, TNR and DNR as well as TPR and DPR almost add up to one, so disrupted contacts account for most separation errors, whereas missed rejections and bycatch remain rare. Under medium flow, the highest TNR reaches 93.5% (1.1 p.p. above FAF) and the highest TPR reaches 96.7% (2.5 p.p. above FAF). The gap widens at high flow: the best TNR rises to 91.3% (2.2 p.p. above FAF) and the best TPR to 92.8% (4.8 p.p. above FAF). Expanding the tree from $N_{R}=N_{B}=2$ to $N_{R}=N_{B}=4$ has little effect on TNR but noticeably improves TPR, especially at high flow (e.g., c=0.5: from 91.2% to 92.8%, a gain of 1.6 p.p.). The cost weight operates as designed: c=0.8 promotes rejection, while c=0.5 favors bycatch avoidance.

With the smaller tree ($N_{R}=N_{B}=2$), median runtimes remain in 2.6 ms to 4.5 ms and worst-case latencies stay below 25 ms at medium flow and below 32 ms at high flow; the larger tree stays moderate at medium flow (3.8 ms to 3.9 ms median, up to 83 ms maximum) but rises sharply to 30.5 ms to 30.7 ms median at high flow (third quartile up to 66 ms, maximum up to 150 ms), making it computationally expensive in dense scenes. MTCR is still several times slower than FAF, which comfortably meets the approximately 10 ms real-time budget; nevertheless, with $N_{R}=N_{B}=2$ it remains in a feasible range, although parallelization as described in Section 6.2 is required to strictly ensure that the real-time constraints are met.

### 7.3. Experimental Settings

We evaluate the laboratory chute-based grid-sorter prototype of Section 6 against a pilot-scale pneumatic high-precision chute sorter (an image of the system can be found, e.g., in [9]). The pneumatic reference uses predictive tracking for nozzle activation at 250 fps and a BAUMER VLXT-50C.I Bayer RGB area-scan camera at up to 2448 x 2048 px over a chute width of 700 mm. The nozzle bar is driven by fast-switching FESTO MHJ10-S-0,35-MF solenoid valves and provides 1000 Hz temporal resolution and about 5.2 mm spatial resolution, with discrete 1 ms trigger intervals. Nozzle expansion was tuned in preliminary experiments to balance successful ejections and bycatch, and the controller parameters were fixed based on preliminary experiments before the reported evaluation runs. For fair comparison, both systems process the same material at similar mass flow and accept-to-reject ratio, with comparable effective chute width on the pneumatic system.

**Figure A3:**
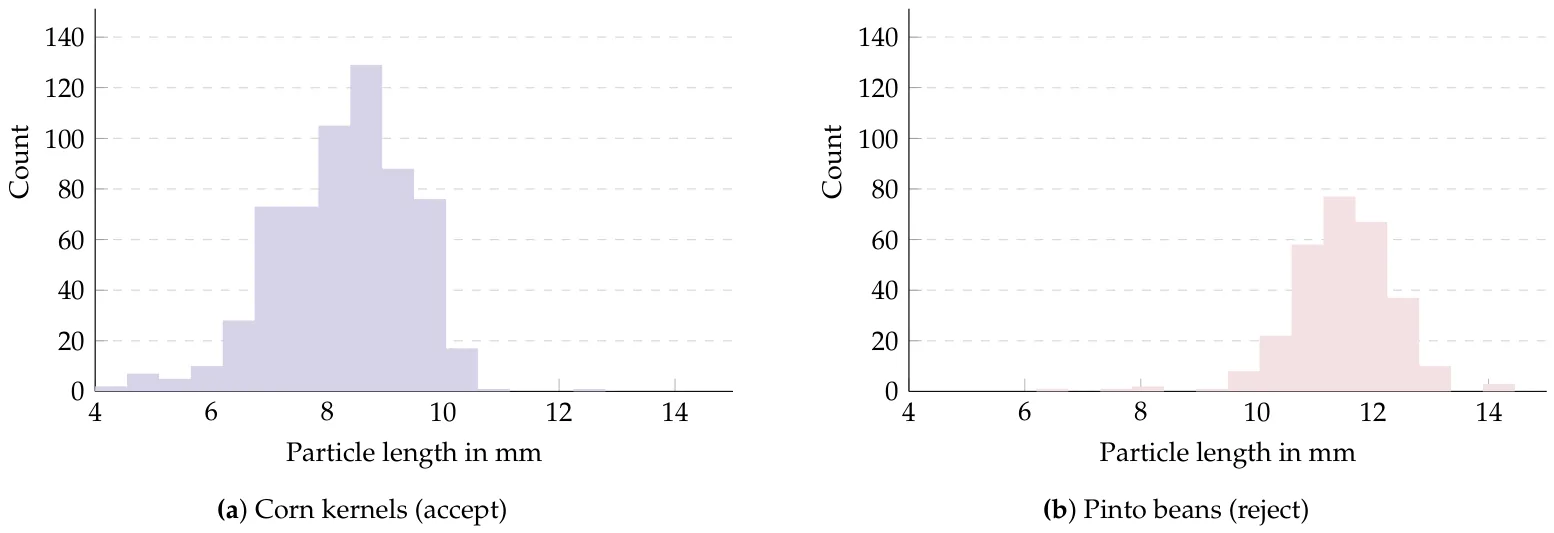
Particle length histograms (longest principal axis) for a representative sorting run of corn kernels (**a**) and pinto beans (**b**).

We conducted five sorting runs of 900 particles each for FAF, for MTCR at each of c∈{0.2,0.5,0.8}, and for the pneumatic reference. The accept-to-reject ratio was 2:1 (corn kernels as accept particles vs. pinto beans as reject particles), with mean particle masses of about 0.19 g (accept) and 0.31 g (reject). The median mass flow was approximately 36 /s particles, corresponding to roughly 6 particles in the grid region or about 242 kg/(hm) across the chute width. This loading is substantially below the simulated scenarios (Section 7.1) due to hardware constraints related to the prototype’s particle feeding system. Consequently, the highest mass flow conditions of this work are demonstrated primarily in simulation. The distributions of particle lengths (longest principal axis) for accept and reject particles from a representative sorting run are shown in Figure A3. Corn kernels had an interquartile range of about 7.6 mm to 9.1 mm and pinto beans of about 11.0 mm to 12.0 mm. The controller again uses $T=t_{\mathrm{cycle}}^{\mathrm{act}}$, with actuator phase durations adapted to the solenoids, but employs a larger horizon of N=15 to accommodate the extended prediction range and velocity variations along the x-axis. To balance runtime and accuracy on the prototype, the tree size is increased to $N_{R}=N_{B}=3$. As in the simulation evaluation, laboratory MTCR runs do not force the standalone FAF sequence into the candidate set. The internal motion model of MTCR and predictive tracking uses CA to account for gravity on the chute.

Although cameras, frame rates, chute geometry, and actuation differ between platforms, several residual differences favor the pneumatic reference relative to the mechanical grid: the pneumatic system operates at a higher frame rate, and particles are not laterally confined by chute side walls as on the prototype. The corn-kernel vs. pinto-bean mixture was chosen so that classification errors are negligible, which keeps the influence of camera differences on the reported sorting metrics small. Therefore, under more closely matched sensing and boundary conditions, we expect the performance gap in favor of the grid to be at least as large.

Energy consumption for separation (excluding cameras, illumination, and the host PC) was assessed in ten dedicated runs under the same settings. On the grid prototype, solenoid electrical energy was measured with a shunt-resistor-based voltage capture and integrated load power over detected activation pulses. For the pneumatic reference, cumulative nozzle-open times were logged and converted to a lower-bound estimate of compressor and valve electrical energy, omitting compressor idle, leaks, and network losses; the estimation procedure is detailed in Appendix H.

### 7.4. Statistical Tests

The analysis of the sorting runs is accompanied by statistical tests of three pre-specified confirmatory alternative hypotheses $H_{1}^{\left(A\right)}$, $H_{1}^{\left(B\right)}$, and $H_{1}^{\left(C\right)}$ on run-level means of TNR and TPR. They reflect the main design features of the sorter architectures and controllers (in principle, further hypotheses could be tested, but statistical power is limited by the small number of runs and additional hypotheses would require substantially more expensive evaluations):

Hypothesis $H_{1}^{\left(A\right)}$. FAF achieves higher TNR than pneumatic predictive tracking, while its TPR is non-inferior. This reflects the grid’s higher effective resolution and sharper impact zones relative to a nozzle bar, while both strategies assign reject particles greedily.

Hypothesis $H_{1}^{\left(B\right)}$. MTCR at c=0.2 achieves higher TPR than pneumatic predictive tracking, while its TNR is non-inferior. This reflects that MTCR explicitly penalizes bycatch and can exploit the grid’s higher resolution. We use c=0.2 as the weight that most strongly penalizes bycatch.

Hypothesis $H_{1}^{\left(C\right)}$. MTCR at c=0.2 achieves higher TPR than FAF on the grid. This reflects that, relative to myopic FAF, MTCR accounts for accept particles; a lower TNR than under FAF is expected.

For $H_{1}^{\left(A\right)}$ and $H_{1}^{\left(B\right)}$, superiority on one metric is required jointly with non-inferiority on the other so that a declared improvement respects the rejection–bycatch trade-off. We call a method non-inferior if a maximum allowed drop of *δ* = 1.5 p.p. relative to the reference is not exceeded [50]. Superiority and non-inferiority are assessed by exact one-sided permutation tests [51] with *p*-values $p_{\sup}^{\left(i\right)}$ and $p_{\mathrm{ni}}^{\left(i\right)}$, respectively, for i∈{A,B,C}; non-inferiority uses a boundary shift by δ. Conjunctions use $p^{\left(i\right)}=\max\left(p_{\sup}^{\left(i\right)},p_{\mathrm{ni}}^{\left(i\right)}\right)$ [52] for i∈{A,B}, and $p^{\left(C\right)}=p_{\sup}^{\left(C\right)}$. Family-wise error over $H_{1}^{\left(A\right)}$–$H_{1}^{\left(C\right)}$ is controlled by Holm–Bonferroni adjustment [53], yielding final values $p_{\mathrm{Holm}}^{\left(i\right)}$ that are compared against α=0.05. We further test the ordered alternative that the mean TPR of MTCR decreases with increasing c∈{0.2,0.5,0.8}, because smaller *c* weights bycatch avoidance more strongly. This trend is assessed with a one-sided Jonckheere–Terpstra test [54,55].

In addition to these statistical tests, we report descriptive statistics for the controllers’ effects on the experimental differences in TNR and TPR relative to pneumatic predictive tracking. For each metric and contrast, we report the difference between the run-level means and a nominal 1−α confidence set obtained by grid-based numerical inversion of the corresponding two-sided permutation test with an exact randomization distribution (see [56]). To this end, for a discretized range of offsets $d_{i}$, the observations of the comparison method are shifted by $-d_{i}$ before a permutation test for equality is evaluated. The nominal confidence set then consists of the $d_{i}$ values, equidistantly spaced by 0.1 p.p., that are not rejected at α=0.05. As a robust alternative, we additionally report the Hodges–Lehmann estimate [57], defined as the median of all pairwise run-level differences, together with a nominal 95% confidence set obtained by grid-based numerical inversion of the corresponding Wilcoxon rank-sum test using its exact permutation distribution [56]. In contrast to the difference in run-level means, the Hodges–Lehmann estimate quantifies the location shift between two empirical distributions. The pairwise statistics are not Holm-adjusted and quantify individual effects; confirmatory significance is assessed only by the tests for $H_{1}^{\left(A\right)}$–$H_{1}^{\left(C\right)}$ and the secondary ordered Jonckheere–Terpstra test.

### 7.5. Experimental Results

**Figure 6:**
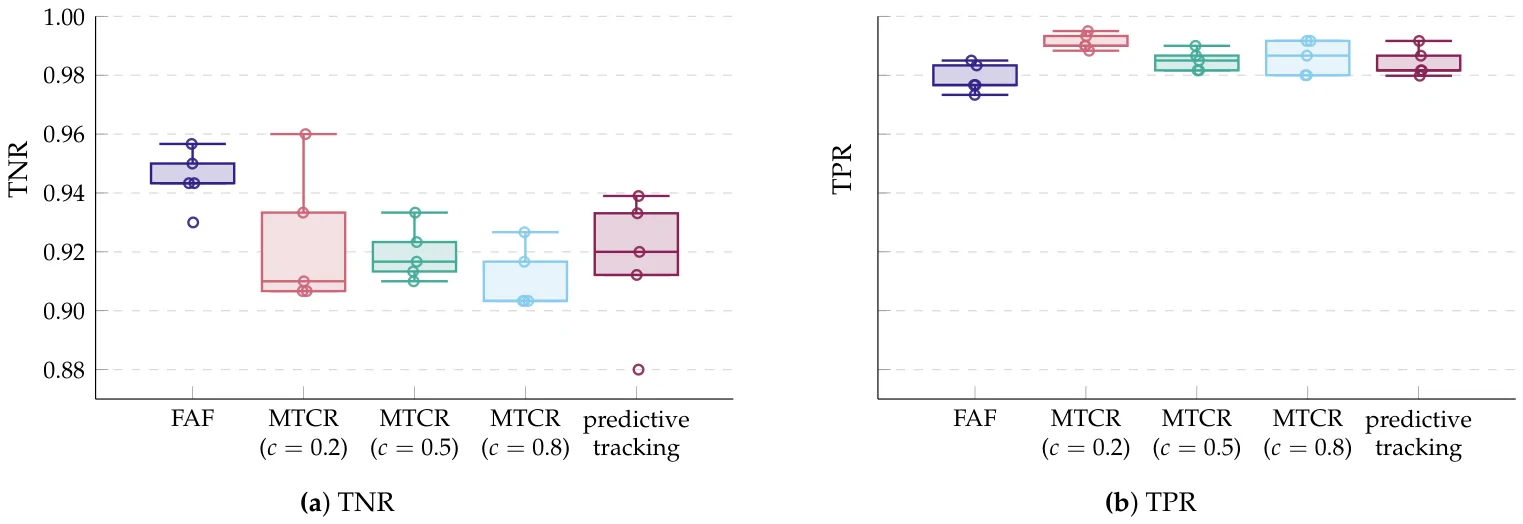
Comparison of the controllers on our prototype grid sorter and of predictive tracking on a high-precision chute sorter, with respect to TNR (**a**) and TPR (**b**). The boxplots summarize the distribution (median, first and third quartiles, with whiskers extending to the most distant data point within 1.5 times the interquartile range), and points show the results of the individual experiments.

Figure 6 summarizes the outcomes; detailed test statistics are given in Appendix I. On the grid prototype, FAF yields the best average TNR (94.5%), followed by MTCR at c=0.2 (92.3%), c=0.5 (91.9%), and c=0.8 (91.0%). The order is inverted for TPR: MTCR at c=0.2 achieves 99.1%, then c=0.8 (98.6%) and c=0.5 (98.5%), each above FAF (97.9%). The corresponding mean accept- and reject-fraction purities (positive and negative predictive values, PPV and NPV) are 97.3%/95.8% for FAF and 96.3%/98.2% for MTCR at c=0.2. The secondary Jonckheere–Terpstra test supports a decreasing TPR trend with increasing *c* (p=0.044), driven chiefly by c=0.2 versus c=0.5. $H_{1}^{\left(C\right)}$ is significant ($p_{\mathrm{Holm}}^{\left(C\right)}=0.012$): MTCR at c=0.2 raises average TPR by 1.2 p.p. over FAF, while average TNR falls by 2.1 p.p.

Relative to pneumatic predictive tracking (91.7% average TNR, 98.4% average TPR; mean PPV/NPV 96.0%/96.7%), $H_{1}^{\left(A\right)}$ is significant ($p_{\mathrm{Holm}}^{\left(A\right)}=0.024$): FAF improves TNR by 2.8 p.p. on average (permutation confidence set: 0.44 p.p. to 5.39 p.p.) with an average TPR deficit of only 0.5 p.p.. The corresponding confidence set for the TPR mean difference is −1.32 p.p.–0.33 p.p.; it thus includes zero while remaining above the prespecified non-inferiority margin of −1.5 p.p.. $H_{1}^{\left(B\right)}$ is not significant ($p_{\mathrm{Holm}}^{\left(B\right)}=0.103$), although MTCR at c=0.2 achieves higher average TPR (0.7 p.p.; confidence set: 0.09 p.p. to 1.33 p.p.) and TNR (0.6 p.p.) than predictive tracking. The broad TNR confidence set, −2.76 p.p. to 4.01 p.p., reflects the uncertainty of this difference.

### 7.6. Comparison of Separation Energy

Table 2 compares electrical energy for separation per run for the grid prototype under FAF (measured) and the pneumatic reference (estimated lower bound). Over the ten runs, the grid consumed on average 57.6 J (±1.9 J), or about 0.192 J per reject particle—close to the theoretical design energy of about 0.15 J per activation. The pneumatic estimate totals 4531 J (±152 J), of which compressed-air generation dominates and valve electrical energy is only about 36 J (consistent with [58], where valve electrical energy was only about 1% of that for generating compressed air). The pneumatic-to-grid energy ratio is about 79, i.e., nearly two orders of magnitude.

## 8. Discussion

The simulation results indicate that the main strength of MTCR is its better handling of the combinatorial assignment problem. This is supported by the larger gains under high mass flow (2.2 p.p. in best-case TNR and 4.8 p.p. in best-case TPR over FAF) compared with the medium-flow improvement (1.1 p.p. in best-case TNR and 2.5 p.p. in best-case TPR). MTCR is therefore most valuable when many closely spaced particles must be coordinated and greedy local choices become inadequate. Enlarging the search tree from $N_{R}=N_{B}=2$ to $N_{R}=N_{B}=4$ mainly improves TPR in dense scenes, at the cost of substantially higher runtime there (30.5 ms to 30.7 ms vs. 4.4 ms to 4.5 ms). In simulation, the weighting parameter *c* provides a practical tuning knob for the rejection–bycatch trade-off: c=0.8 favors rejection slightly, whereas c=0.5 favors bycatch avoidance. In the laboratory, this trend appears mainly on the TPR side and is significant in a secondary analysis (c=0.2 yields the highest average TPR with 99.1%). However, TNR remains highest under FAF and shows no clear increase with *c* among the MTCR variants.

The main weakness of MTCR is its runtime (2.6 ms to 4.5 ms with $N_{R}=N_{B}=2$ vs. sub-millisecond latency for FAF), and the experiments suggest that this penalty itself degrades accuracy. In simulation, where computation delay does not affect the applied control, MTCR clearly outperforms FAF. In the laboratory, however, FAF reaches higher average TNR (94.5% vs. 92.3% at c=0.2), even though MTCR yields a significantly higher TPR (99.1% vs. 97.9%; $H_{1}^{\left(C\right)}$). Because both controllers rely on the same predicted arrival times, the most plausible explanation is the greater age of MTCR decisions when they are applied. Runtime optimization is therefore a direct accuracy lever and a major direction for further development.

Relative to the pneumatic reference, FAF significantly increases rejection performance at comparable bycatch ($H_{1}^{\left(A\right)}$). Although $H_{1}^{\left(B\right)}$ does not reach significance, likely because the test is underpowered for the modest effect sizes involved, MTCR at c=0.2 increases average TPR by 0.7 p.p., suggesting that the higher grid resolution may indeed help to reduce bycatch. In summary, the experiments demonstrate precise separation without compressed air: on the prototype, FAF achieves an average TNR that is 2.8 p.p. higher than the pneumatic reference (94.5% vs. 91.7%)—a result that exceeds the state of the art despite early prototype-level hardware—while actuation energy is lower by at least a factor of 79 in our application.

## 9. Conclusions

This work presented a next-generation sensor-based sorting concept that moves beyond the long-established pneumatic ejection paradigm of one-dimensional nozzle bars on belts and chutes. We proposed two central novelties: (i) a two-dimensional actuator grid that replaces the conventional one-dimensional nozzle row and increases effective spatio-temporal resolution through redundant particle–actuator pairings; and (ii) impact-based mechanical actuators within the grid that avoid compressed air entirely, cutting energy usage by one to two orders of magnitude and eliminating a major operating-cost driver.

To coordinate ejection online under partial observability, we formulated sorting as a finite-horizon SOC problem and introduced the MTCR controller. It combines physically motivated action-space reduction, OLF evaluation of expected sorting costs, mixed discrete–continuous-time modeling, and pruned tree search completed by a greedy FAF rollout, applied in a receding-horizon manner.

Simulation shows that MTCR improves rejection and avoidance of bycatch over FAF, with the largest gains under high mass flow where combinatorial assignment dominates; enlarging the search tree mainly improves TPR in dense scenes, and a weighting parameter offers a practical rejection–bycatch trade-off in simulation. The main open point is runtime (2.6 ms to 4.5 ms per step with $N_{R}=N_{B}=2$, and 30.5 ms to 30.7 ms for the expanded tree at high flow, vs. sub-millisecond latency for FAF), which may help explain why FAF achieves higher experimental TNR despite identical predicted arrival times—and thus represents a direct path to higher field performance. Additionally, prototype particle feeding system limitations restricted high mass flow evaluations to simulation, leaving physical experiments under moderate loading conditions, albeit with a high 2:1 accept-to-reject ratio. Regarding the experimental results, FAF on the grid significantly exceeds the rejection performance of a pneumatic high-precision chute sorter by 2.8 p.p. in TNR with non-inferior TPR, and MTCR at c=0.2 yields a further statistically significant improvement in TPR over FAF on the same hardware while trading off some TNR—results that already advance the state of the art despite the early development stage.

Overall, pairing higher effective resolution with substantially lower energy demand makes the proposed architecture a strong candidate for cost-sensitive, high-throughput sorting—notably for applications lacking pneumatic infrastructure and for sectors under economic pressure, such as recycling, where operating costs constrain today’s systems.

## 10. Patents

Parts of this work are disclosed in the European patent EP4309807B1 [59] and corresponding patent applications in further regions. The patent covers the grid-sorter concept, control approaches ranging from simple assignment heuristics to general references to solution paradigms such as MPC and reinforcement learning, and ideas not presented here, such as grid architectures with pneumatic nozzles, multi-fraction sorting, and coordinated multi-actuator ejection. The grid of impact-based actuators presented here is one advantageous special case of this broader architecture. The concrete MTCR controller developed and evaluated in this article goes beyond the patent disclosure.

## Figures and Tables

**Table 1:** Sorting results and per-step controller runtime (median/third quartile/maximum latency) for medium and high mass flow. Bold values indicate the best results in each column.

	Medium Mass Flow			High Mass Flow		
	**TNR/TPR** **in %**	**DNR/DPR** **in %**	**Runtime** **in ms**	**TNR/TPR** **in %**	**DNR/DPR** **in %**	**Runtime** **in ms**
MTCR						
c=0.5, $N_{R}=N_{B}=2$	93.3/96.2	6.2/3.5	2.7/3.2/22.6	91.2/91.2	8.3/8.1	4.5/4.9/31.8
c=0.8, $N_{R}=N_{B}=2$	93.4/96.0	6.2/3.7	2.6/3.1/24.2	**91.3**/90.9	8.3/8.3	4.4/5.1/28.8
c=0.5, $N_{R}=N_{B}=4$	**93.5**/**96.7**	**5.9**/ **3.2**	3.9/12.0/82.5	91.0/**92.8**	**8.2**/**6.9**	30.5/65.3/148.3
c=0.8, $N_{R}=N_{B}=4$	**93.5**/96.5	6.1/3.3	3.8/11.8/81.8	**91.3**/92.3	8.3/7.3	30.7/65.7/150.1
FAF	92.4/94.2	7.3/4.8	**0.5**/**0.6**/**1.5**	89.1/88.0	10.5/10.1	**0.7**/**0.9**/**2.5**

**Table 2:** Electrical energy for separation per sorting run (mean ± sample standard deviation over ten runs). Per-particle quantities divide by 300 reject particles.

Quantity	Mechanical Grid (Measured)	Pneumatic (Estimated)
Compressed-air generation	—	4495 ± 151 J
Actuator/valve electrical	57.6 ± 1.9. J	36.2 ± 0.8 J
Total	57.6 ± 1.9. J	4531 ± 152 J
Total per reject particle	0.192 J	15.1 J

**Figure A1:**
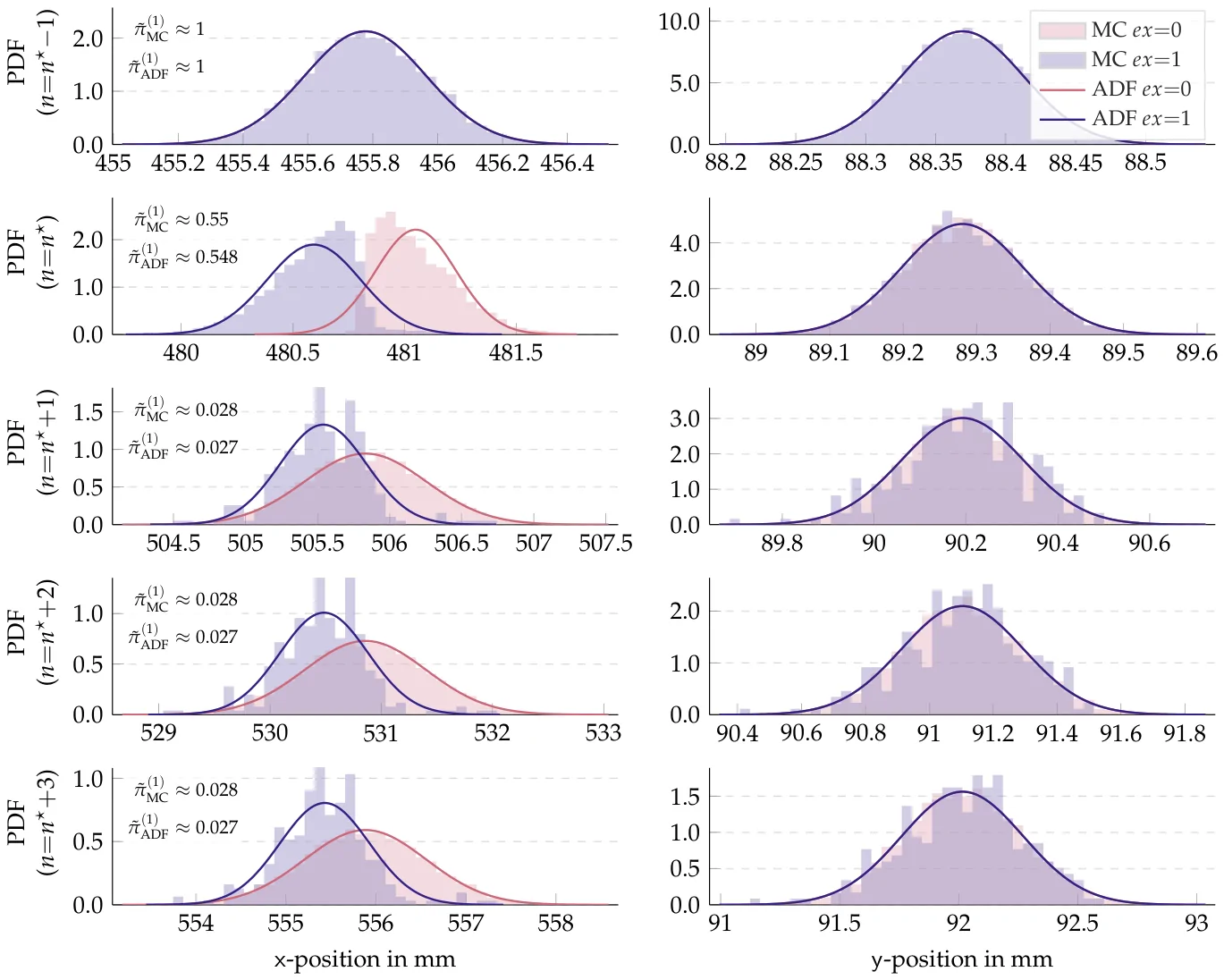
Monte Carlo and ADF-predicted position densities for an example particle with contact at stage $n^{★}=2$ by the actuator at (465 mm, 91.5 mm) (width 9 mm). The track had 20 associated measurements before prediction. Histograms show empirical Monte Carlo distributions for ex∈{0,1}, solid curves show ADF Gaussians. Annotations report predicted existence probabilities ${\tilde{\pi }}_{\mathrm{MC}}^{\left(1\right)}$ (fraction of samples not yet ejected) and ${\tilde{\pi }}_{\mathrm{ADF}}^{\left(1\right)}$.

**Figure A2:**
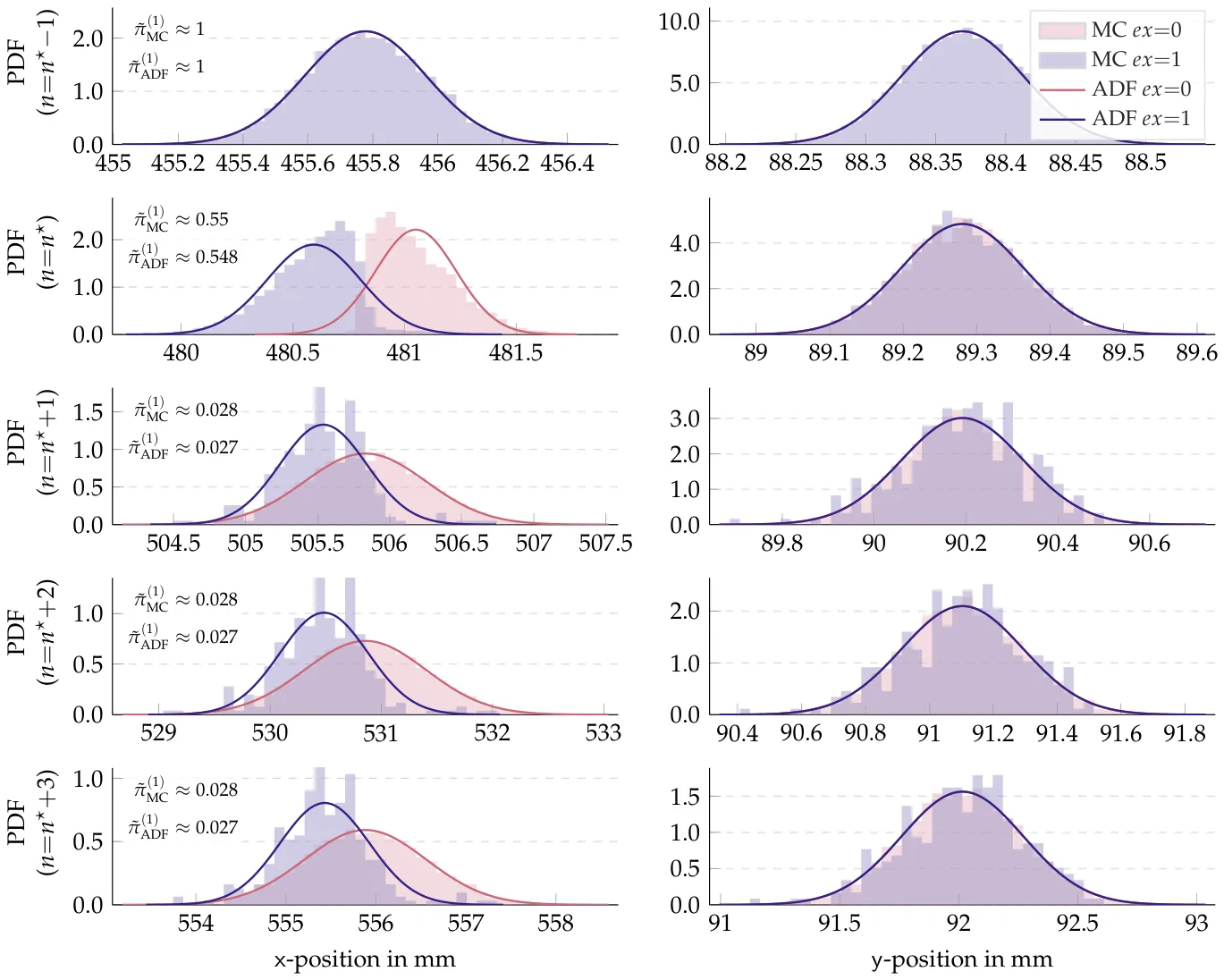
Monte Carlo and ADF-predicted position densities for an example particle with contact at stage $n^{★}=2$ by the actuator at (585 mm, 39.5 mm) (width 9 mm). The track had 22 associated measurements before prediction. Histograms show empirical Monte Carlo distributions for ex∈{0,1}, solid curves show ADF Gaussians. Annotations report predicted existence probabilities ${\tilde{\pi }}_{\mathrm{MC}}^{\left(1\right)}$ (fraction of samples not yet ejected) and ${\tilde{\pi }}_{\mathrm{ADF}}^{\left(1\right)}$.

## Data Availability

The original data presented in the study are openly available in GitHub at https://github.com/KIT-ISAS/2DArraySorting_SNMPC/tree/v1.0.1 (accessed on 19 August 2026).

## Appendix A. Forward and Backward Actuator Dynamics

We will now derive the mathematical form of the continuous-time actuator dynamics ${\tilde{f}}^{\mathrm{act}}:\left[0,T\right]\times \left[0,t_{\mathrm{cycle}}^{\mathrm{act}}\right)\times U\to \left[0,t_{\mathrm{cycle}}^{\mathrm{act}}\right)\;,\;\left(t,t_{k-1}^{\mathrm{act}}\left(T\right),u_{k}\right)\mapsto t^{\mathrm{act}}$ and its inverse ${\tilde{g}}^{\mathrm{act}}:\left(0,t_{\mathrm{cycle}}^{\mathrm{act}}\right)\times \left[0,t_{\mathrm{cycle}}^{\mathrm{act}}\right)\times U\to \left[0,T\right)\cup \left\{\infty \right\}\;,\;\left(t^{\mathrm{act}},t_{k-1}^{\mathrm{act}}\left(T\right),u_{k}\right)\mapsto t$.

### Appendix A.1. Forward Actuator Dynamics

${\tilde{f}}^{\mathrm{act}}\left(\cdot \right)$ has a sawtooth profile with linear and constant (only at $t^{\mathrm{act}}=0$) segments (see Figure 2). Since time advances equally for both *t* and $t^{\mathrm{act}}$ (both axes in Figure 2), the slope of the linear segments is 1. Three cases need to be distinguished: (i) the actuator movement is caused by a previous activation (via $t_{k-1}^{\mathrm{act}}\left(T\right)$), (ii) the actuator movement is caused by an activation in the current time step (via $u_{k}$), or (iii) there is no actuator movement. For each of the cases, we will now derive the corresponding relation and condition.

Case (i) actuator movement due to a previous activation:

$$\begin{array}{c} t^{\mathrm{act}}=t+t_{k-1}^{\mathrm{act}}\left(T\right)\;\mathrm{if}\;t_{k-1}^{\mathrm{act}}\left(T\right)>0\;\mathrm{and}\;t+t_{k-1}^{\mathrm{act}}\left(T\right)<t_{\mathrm{cycle}}^{\mathrm{act}}\;. \end{array}$$

Case (ii) actuator movement due to an activation in the current time step:

$$\begin{array}{cc} t^{\mathrm{act}}=t-u_{k}\;\mathrm{if}\; & 0\leq u_{k}\leq t\\ \; & \mathrm{and}\left(u_{k}\geq t_{\mathrm{cycle}}^{\mathrm{act}}-t_{k-1}^{\mathrm{act}}\left(T\right)\;\mathrm{if}\;\;t_{k-1}^{\mathrm{act}}\left(T\right)>0\right)\;. \end{array}$$

The first condition describes the time range in which the line segment is affected by $u_{k}$, while the second ensures that $u_{k}$ is ignored if the actuator is still moving from a previous activation.

Case (iii) no actuator movement:

$$\begin{array}{c} t^{\mathrm{act}}=0\;\mathrm{if}\;\mathrm{neither}\;\mathrm{case}\;\left(i\right)\;\mathrm{nor}\;\mathrm{case}\;\left(\mathrm{ii}\right)\;\mathrm{applies}. \end{array}$$

This case is ambiguous since $t^{\mathrm{act}}=0$ for a wide range of *t*, $t_{k-1}^{\mathrm{act}}\left(T\right)$, and $u_{k}$.

### Appendix A.2. Inverse Actuator Dynamics

The three cases from the forward dynamics carry over as follows:

Case (i) actuator movement due to a previous activation:

$$\begin{array}{c} t=t^{\mathrm{act}}-t_{k-1}^{\mathrm{act}}\left(T\right)\;\mathrm{if}\;t_{k-1}^{\mathrm{act}}\left(T\right)>0\;\mathrm{and}\;t_{k-1}^{\mathrm{act}}\left(T\right)\leq t^{\mathrm{act}}\;\mathrm{and}\;t^{\mathrm{act}}-t_{k-1}^{\mathrm{act}}\left(T\right)<T\;. \end{array}$$

The second and third conditions ensure that $t^{\mathrm{act}}\left(t\right)$ is reached within the current time step.

Case (ii) actuator movement due to an activation in the current time step:

$$\begin{array}{cc} t=t^{\mathrm{act}}+u_{k}\;\mathrm{if}\; & 0\leq u_{k}<t^{\mathrm{act}}\\ \; & \mathrm{and}\;\left(u_{k}\geq t_{\mathrm{cycle}}^{\mathrm{act}}-t_{k-1}^{\mathrm{act}}\left(T\right)\;\mathrm{if}\;\;t_{k-1}^{\mathrm{act}}\left(T\right)>0\right)\;. \end{array}$$

Analogously to the forward dynamics, the first condition specifies the internal actuator time range in which the line segment is affected by $u_{k}$, while the second ensures that $u_{k}$ is ignored if the actuator is still moving from a previous activation.

Case (iii) no actuator movement:

$$\begin{array}{c} t=\infty \;\mathrm{if}\;\mathrm{neither}\;\mathrm{case}\;\left(i\right)\;\mathrm{nor}\;\mathrm{case}\;\left(\mathrm{ii}\right)\;\mathrm{applies}. \end{array}$$

## Appendix B. Moment Matching in ADF-Inspired Prediction for Multiple Disjoint Threshold Conditions

We derive the moment-matching step of ADF-inspired prediction (Section 5.3) for a slightly simplified hybrid transition compared with Section 5.2. As in the main text, the particle index is omitted. Intermediate quantities attached to ${\underline{x}}_{k-1}^{\mathrm{mot}}$ are written without an explicit time subscript on the symbol when it is clear from context. The discrete transition is a product of $N_{A}$ linear indicator conditions,

$$\begin{array}{c} P\;\left({\mathrm{ex}}_{k}=0|{\underline{x}}_{k-1}^{\mathrm{mot}},ex_{k-1}=s\right)=1-\prod_{j=1}^{N_{A}}\left(1-1_{S_{k-1}^{\left(j,s\right)}}\left(H_{k-1}^{\left(j,s\right)}{\underline{x}}_{k-1}^{\mathrm{mot}}+{\underline{d}}_{k-1}^{\left(j,s\right)}\right)\right)\;, \end{array}$$

where $H_{k-1}^{\left(j,s\right)}\in R^{n_{\underline{\xi }}\times n_{\underline{x}}}$ and ${\underline{d}}_{k-1}^{\left(j,s\right)}\in R^{n_{\underline{\xi }}}$ with $n_{\underline{\xi }}\leq n_{\underline{x}}$ are the linear map and offset of the *j*-th subcondition. We assume throughout that the sets $S_{k-1}^{\left(j,s\right)}$ are pairwise disjoint.

### Appendix B.1. Geometric Interpretation

The product encodes a logical OR (union) of $N_{A}$ linear subconditions. With the state regions

$$\begin{array}{c} R_{k-1}^{\left(j,s\right)}=\left\{{\underline{x}}_{k-1}^{\mathrm{mot}}\in R^{n_{\underline{x}}}\;|\;H_{k-1}^{\left(j,s\right)}{\underline{x}}_{k-1}^{\mathrm{mot}}+{\underline{d}}_{k-1}^{\left(j,s\right)}\in S_{k-1}^{\left(j,s\right)}\right\}\;, \end{array}$$

one has

$$\begin{array}{c} P\;\left({\mathrm{ex}}_{k}=0|{\underline{x}}_{k-1}^{\mathrm{mot}},ex_{k-1}=s\right)=1_{{⋃}_{j=1}^{N_{A}}R_{k-1}^{\left(j,s\right)}}\left({\underline{x}}_{k-1}^{\mathrm{mot}}\right)\;. \end{array}$$

Evaluating the indicator of a general union requires inclusion–exclusion with complexity $O\left(2^{N_{A}}\right)$. Under pairwise disjointness of the sets $S_{k-1}^{\left(j,s\right)}$—and hence of the regions $R_{k-1}^{\left(j,s\right)}$—the union indicator reduces to the sum

$$\begin{array}{c} 1_{{⋃}_{j=1}^{N_{A}}R_{k-1}^{\left(j,s\right)}}\left({\underline{x}}_{k-1}^{\mathrm{mot}}\right)=\sum_{j=1}^{N_{A}}1_{S_{k-1}^{\left(j,s\right)}}\left(H_{k-1}^{\left(j,s\right)}{\underline{x}}_{k-1}^{\mathrm{mot}}+{\underline{d}}_{k-1}^{\left(j,s\right)}\right)\;, \end{array}$$

of complexity $O\left(N_{A}\right)$. After threshold evaluation, the unnormalized intermediate density for mode r=0 is therefore

(A1) $$\begin{array}{c} q^{\left(0\right)}\left({\underline{x}}_{k-1}^{\mathrm{mot}}\right)=\sum_{s\in \{0,1\}}{\tilde{\pi }}_{k-1}^{\left(s\right)}\sum_{j=1}^{N_{A}}N\left({\underline{x}}_{k-1}^{\mathrm{mot}};{\underline{\mu }}_{k-1}^{\left(s\right)},P_{k-1}^{\left(s\right)}\right)1_{S_{k-1}^{\left(j,s\right)}}\left(H_{k-1}^{\left(j,s\right)}{\underline{x}}_{k-1}^{\mathrm{mot}}+{\underline{d}}_{k-1}^{\left(j,s\right)}\right)\;. \end{array}$$

Moment matching thus requires the moments of the truncated components

$$\begin{array}{c} q_{j}^{\left(s\right)}\left({\underline{x}}_{k-1}^{\mathrm{mot}}\right)=N\left({\underline{x}}_{k-1}^{\mathrm{mot}};{\underline{\mu }}_{k-1}^{\left(s\right)},P_{k-1}^{\left(s\right)}\right)1_{S_{k-1}^{\left(j,s\right)}}\left(H_{k-1}^{\left(j,s\right)}{\underline{x}}_{k-1}^{\mathrm{mot}}+{\underline{d}}_{k-1}^{\left(j,s\right)}\right) \end{array}$$

for all $j=1,\ldots ,N_{A}$ and s∈{0,1}. Introducing the auxiliary variable ${\underline{\xi }}^{\left(j,s\right)}=H_{k-1}^{\left(j,s\right)}{\underline{x}}_{k-1}^{\mathrm{mot}}+{\underline{d}}_{k-1}^{\left(j,s\right)}$, one has $p\;\left({\underline{x}}_{k-1}^{\mathrm{mot}}|{\underline{\xi }}^{\left(j,s\right)}\in S_{k-1}^{\left(j,s\right)}\right)\propto q_{j}^{\left(s\right)}\left({\underline{x}}_{k-1}^{\mathrm{mot}}\right)$, with normalizing mass

$$\begin{array}{c} w_{j}^{\left(s\right)}=\int_{S_{k-1}^{\left(j,s\right)}}N\left(\underline{\xi };{\underline{\mu }}_{\underline{\xi }}^{\left(j,s\right)},P_{\underline{\xi }}^{\left(j,s\right)}\right)\;d\;\underline{\xi } \end{array}$$

equal to the prior probability mass of mode *s* inside the threshold region $S_{k-1}^{\left(j,s\right)}$.

### Appendix B.2. Gaussian Forward and Backward Projection

In the linear-Gaussian setting, the moments of $p\;\left({\underline{x}}_{k-1}^{\mathrm{mot}}|{\underline{\xi }}^{\left(j,s\right)}\in S_{k-1}^{\left(j,s\right)}\right)$ are obtained by projecting between the full state space $R^{n_{\underline{x}}}$ and the lower-dimensional subspace associated with $S_{k-1}^{\left(j,s\right)}\subseteq R^{n_{\underline{\xi }}}$. The law of total expectation and variance yields the general expressions

$$\begin{array}{cc} {\underline{\mu }}_{\underline{x},j}^{\left(s\right)} & =E\;\left\{{\underline{x}}_{k-1}^{\mathrm{mot}}|{\underline{\xi }}^{\left(j,s\right)}\in S_{k-1}^{\left(j,s\right)}\right\}=E\;\left\{E\;\left\{{\underline{x}}_{k-1}^{\mathrm{mot}}|{\underline{\xi }}^{\left(j,s\right)}\right\}|{\underline{\xi }}^{\left(j,s\right)}\in S_{k-1}^{\left(j,s\right)}\right\}\;,\\ P_{\underline{x},j}^{\left(s\right)} & =\mathrm{Var}\;\left\{{\underline{x}}_{k-1}^{\mathrm{mot}}|{\underline{\xi }}^{\left(j,s\right)}\in S_{k-1}^{\left(j,s\right)}\right\}\\ & =E\;\left\{\mathrm{Var}\;\left\{{\underline{x}}_{k-1}^{\mathrm{mot}}|{\underline{\xi }}^{\left(j,s\right)}\right\}|{\underline{\xi }}^{\left(j,s\right)}\in S_{k-1}^{\left(j,s\right)}\right\}+\mathrm{Var}\;\left\{E\;\left\{{\underline{x}}_{k-1}^{\mathrm{mot}}|{\underline{\xi }}^{\left(j,s\right)}\right\}|{\underline{\xi }}^{\left(j,s\right)}\in S_{k-1}^{\left(j,s\right)}\right\} \end{array}$$

for the conditional moments. To this end, one first needs the untruncated conditional mean and covariance of ${\underline{x}}_{k-1}^{\mathrm{mot}}$ given ${\underline{\xi }}^{\left(j,s\right)}$. Since ${\underline{x}}_{k-1}^{\mathrm{mot}}$ and ${\underline{\xi }}^{\left(j,s\right)}$ are jointly Gaussian before truncation,

$$\begin{array}{cc} E\;\left\{{\underline{x}}_{k-1}^{\mathrm{mot}}|{\underline{\xi }}^{\left(j,s\right)}\right\} & ={\underline{\mu }}_{k-1}^{\left(s\right)}+K^{\left(j,s\right)}\left({\underline{\xi }}^{\left(j,s\right)}-{\underline{\mu }}_{\underline{\xi }}^{\left(j,s\right)}\right)\;,\\ \mathrm{Var}\;\left\{{\underline{x}}_{k-1}^{\mathrm{mot}}|{\underline{\xi }}^{\left(j,s\right)}\right\} & =P_{k-1}^{\left(s\right)}-K^{\left(j,s\right)}P_{\underline{\xi }}^{\left(j,s\right)}{\left(K^{\left(j,s\right)}\right)}^{⊤}\;, \end{array}$$

with forward moments and projection gain

$$\begin{array}{cc} {\underline{\mu }}_{\underline{\xi }}^{\left(j,s\right)} & =H_{k-1}^{\left(j,s\right)}{\underline{\mu }}_{k-1}^{\left(s\right)}+{\underline{d}}_{k-1}^{\left(j,s\right)}\;,\\ P_{\underline{\xi }}^{\left(j,s\right)} & =H_{k-1}^{\left(j,s\right)}P_{k-1}^{\left(s\right)}{\left(H_{k-1}^{\left(j,s\right)}\right)}^{⊤}\;,\\ K^{\left(j,s\right)}\; & =P_{k-1}^{\left(s\right)}{\left(H_{k-1}^{\left(j,s\right)}\right)}^{⊤}{\left(P_{\underline{\xi }}^{\left(j,s\right)}\right)}^{-1}\;. \end{array}$$

The truncated moments then read

$$\begin{array}{cc} {\underline{\mu }}_{\underline{x},j}^{\left(s\right)} & ={\underline{\mu }}_{k-1}^{\left(s\right)}+K^{\left(j,s\right)}\left({\underline{\mu }}_{y,\mathrm{trunc}}^{\left(j,s\right)}-{\underline{\mu }}_{\underline{\xi }}^{\left(j,s\right)}\right)\;,\\ P_{\underline{x},j}^{\left(s\right)} & =P_{k-1}^{\left(s\right)}+K^{\left(j,s\right)}\left(P_{y,\mathrm{trunc}}^{\left(j,s\right)}-P_{\underline{\xi }}^{\left(j,s\right)}\right){\left(K^{\left(j,s\right)}\right)}^{⊤}\;, \end{array}$$

where ${\underline{\mu }}_{y,\mathrm{trunc}}^{\left(j,s\right)}$ and $P_{y,\mathrm{trunc}}^{\left(j,s\right)}$ are the mean and covariance of the truncated Gaussian ${\mathrm{TN}}_{S_{k-1}^{\left(j,s\right)}}\left({\underline{\mu }}_{\underline{\xi }}^{\left(j,s\right)},P_{\underline{\xi }}^{\left(j,s\right)}\right)$.

### Appendix B.3. Final Composed Moments

The unnormalized moments—mass $M_{0}^{\left(r\right)}$, first moment $M_{1}^{\left(r\right)}$, and second moment $M_{2}^{\left(r\right)}$—must be obtained for both modes r∈{0,1}. For mode r=0, summing over subconditions and prior modes in Equation (A1) yields

$$\begin{array}{cc} M_{0}^{\left(r=0\right)} & =\sum_{s\in \{0,1\}}{\tilde{\pi }}_{k-1}^{\left(s\right)}\sum_{j=1}^{N_{A}}w_{j}^{\left(s\right)}\;,\\ M_{1}^{\left(r=0\right)} & =\sum_{s\in \{0,1\}}{\tilde{\pi }}_{k-1}^{\left(s\right)}\sum_{j=1}^{N_{A}}w_{j}^{\left(s\right)}{\underline{\mu }}_{\underline{x},j}^{\left(s\right)}\;,\\ M_{2}^{\left(r=0\right)} & =\sum_{s\in \{0,1\}}{\tilde{\pi }}_{k-1}^{\left(s\right)}\sum_{j=1}^{N_{A}}w_{j}^{\left(s\right)}\left[P_{\underline{x},j}^{\left(s\right)}+{\underline{\mu }}_{\underline{x},j}^{\left(s\right)}{\left({\underline{\mu }}_{\underline{x},j}^{\left(s\right)}\right)}^{⊤}\right]\;. \end{array}$$

For the complementary mode r=1, truncation over the non-convex complement $R^{n_{\underline{\xi }}}\setminus {⋃}_{j=1}^{N_{A}}S_{k-1}^{\left(j,s\right)}$ is avoided via

$$\begin{array}{c} M_{q}^{\left(r=1\right)}=M_{q,\mathrm{prior}}-M_{q}^{\left(r=0\right)}\;\mathrm{for}\;q\in \left\{0,1,2\right\}\;, \end{array}$$

which follows from $q^{\left(0\right)}+q^{\left(1\right)}$ recovering the prior mixture of scaled Gaussians. The prior moments are

$$\begin{array}{cc} M_{0,\mathrm{prior}} & =\sum_{s\in \{0,1\}}{\tilde{\pi }}_{k-1}^{\left(s\right)}\;,\\ M_{1,\mathrm{prior}} & =\sum_{s\in \{0,1\}}{\tilde{\pi }}_{k-1}^{\left(s\right)}{\underline{\mu }}_{k-1}^{\left(s\right)}\;,\\ M_{2,\mathrm{prior}} & =\sum_{s\in \{0,1\}}{\tilde{\pi }}_{k-1}^{\left(s\right)}\left[P_{k-1}^{\left(s\right)}+{\underline{\mu }}_{k-1}^{\left(s\right)}{\left({\underline{\mu }}_{k-1}^{\left(s\right)}\right)}^{⊤}\right]\;. \end{array}$$

Normalizing by the mass $M_{0}^{\left(r\right)}$ recovers the scaled-Gaussian parameters

$$\begin{array}{c} {\tilde{\pi }}_{k}^{\left(r\right)}=M_{0}^{\left(r\right)}\;,\;{\tilde{\underline{\mu }}}_{k-1}^{\left(r\right)}=\frac{1}{{\tilde{\pi }}_{k}^{\left(r\right)}}M_{1}^{\left(r\right)}\;,\;{\tilde{P}}_{k-1}^{\left(r\right)}=\frac{1}{{\tilde{\pi }}_{k}^{\left(r\right)}}M_{2}^{\left(r\right)}-{\tilde{\underline{\mu }}}_{k-1}^{\left(r\right)}{\left({\tilde{\underline{\mu }}}_{k-1}^{\left(r\right)}\right)}^{⊤}\;. \end{array}$$

## Appendix C. Moment Matching in ADF-Inspired Prediction for the Particle and Contact Model

We now specialize Appendix B to the particle and contact model of Section 5.2. As before, the particle index *i* is omitted. In contrast to hard indicator products, ejection at actuator $j\in \left\{1,\ldots ,N_{A}\right\}$ during phase $m\in \Omega_{\mathrm{in}}=\left\{\mathrm{HIT},\mathrm{UP},\mathrm{DOWN}\right\}$ is Bernoulli with parameter $p_{\mathrm{eject},m}\in \left[0,1\right]$, and the transition depends asymmetrically on the previous existence mode, according to

$$\begin{array}{c} P\;\left({\mathrm{ex}}_{k}=0|{\underline{x}}_{k-1}^{\mathrm{mot}},ex_{k-1}=0\right)=1\;,\\ P\;\left({\mathrm{ex}}_{k}=0|{\underline{x}}_{k-1}^{\mathrm{mot}},ex_{k-1}=1\right)=1-\prod_{j=1}^{N_{A}}\left(1-\sum_{m\in \Omega_{\mathrm{in}}}p_{\mathrm{eject},m}\;\cdot \;1_{S_{k-1}^{\left(j,m\right)}}\left(H_{k-1}^{\left(j\right)}{\underline{x}}_{k-1}^{\mathrm{mot}}+{\underline{d}}_{k-1}^{\left(j\right)}\right)\right)\;. \end{array}$$

Here, $S_{k-1}^{\left(j,m\right)}\subset R^{2}$ is the spatio-temporal box in time *t* and lateral coordinate y, while $H_{k-1}^{\left(j\right)}$ and ${\underline{d}}_{k-1}^{\left(j\right)}$ follow from the Gauß–Taylor approximations.

### Appendix C.1. Geometric Interpretation

As in Appendix B, the product encodes a logical OR over actuator contacts. To reduce the union indicator to a sum, the boxes $S_{k-1}^{\left(j,m\right)}$ must be pairwise disjoint for all $\left(j_{1},m_{1}\right)\neq \left(j_{2},m_{2}\right)$. Each box is a rectangle in the (t,y)-plane, defined by the start of phase *m* and its duration for actuator *j* (determined by the control sequence), and the fixed lateral hit region of actuator *j*. Within each row, the boxes are disjoint because actuators do not overlap. Across rows, disjointness is guaranteed whenever the activation times of laterally overlapping actuators differ by at least $t_{\mathrm{cycle}}^{\mathrm{act}}$. For typical grid geometries and particle speeds, this holds in the vast majority of configurations; the only possible exception is laterally overlapping actuators in neighboring rows that are activated at similar times. Since MTCR assigns each particle to at most one actuator, such near-simultaneous activations can only arise from two or more closely neighboring particles, and we neglect contributions from the typically small overlaps (compared with the box sizes) of such events in the following. With

$$\begin{array}{c} R_{k-1}^{\left(j,m\right)}=\left\{{\underline{x}}_{k-1}^{\mathrm{mot}}\in R^{n_{\underline{x}}}\;|\;H_{k-1}^{\left(j\right)}{\underline{x}}_{k-1}^{\mathrm{mot}}+{\underline{d}}_{k-1}^{\left(j\right)}\in S_{k-1}^{\left(j,m\right)}\right\}\;, \end{array}$$

the living-mode transition reduces to the soft-weighted sum

$$\begin{array}{c} P\;\left({\mathrm{ex}}_{k}=0|{\underline{x}}_{k-1}^{\mathrm{mot}},ex_{k-1}=1\right)=\sum_{j=1}^{N_{A}}\sum_{m\in \Omega_{\mathrm{in}}}p_{\mathrm{eject},m}\;\cdot \;1_{R_{k-1}^{\left(j,m\right)}}\left({\underline{x}}_{k-1}^{\mathrm{mot}}\right)\;. \end{array}$$

Together with $P\;\left({\mathrm{ex}}_{k}=0|{\underline{x}}_{k-1}^{\mathrm{mot}},ex_{k-1}=0\right)=1$, the intermediate density for mode r=0 becomes

(A2) $$\begin{array}{cc} q^{\left(0\right)}\left({\underline{x}}_{k-1}^{\mathrm{mot}}\right) & ={\tilde{\pi }}_{k-1}^{\left(0\right)}N\left({\underline{x}}_{k-1}^{\mathrm{mot}};{\underline{\mu }}_{k-1}^{\left(0\right)},P_{k-1}^{\left(0\right)}\right)\\ & \;+{\tilde{\pi }}_{k-1}^{\left(1\right)}\sum_{j=1}^{N_{A}}\sum_{m\in \Omega_{\mathrm{in}}}p_{\mathrm{eject},m}\;N\left({\underline{x}}_{k-1}^{\mathrm{mot}};{\underline{\mu }}_{k-1}^{\left(1\right)},P_{k-1}^{\left(1\right)}\right)1_{S_{k-1}^{\left(j,m\right)}}\left(H_{k-1}^{\left(j\right)}{\underline{x}}_{k-1}^{\mathrm{mot}}+{\underline{d}}_{k-1}^{\left(j\right)}\right)\;. \end{array}$$

Thus, mode s=0 contributes its entire prior component, whereas mode s=1 contributes only soft-weighted truncations onto the actuator–phase boxes. The truncated masses and projected moments of the living-mode terms

$$\begin{array}{c} q_{j,m}^{\left(1\right)}\left({\underline{x}}_{k-1}^{\mathrm{mot}}\right)=N\left({\underline{x}}_{k-1}^{\mathrm{mot}};{\underline{\mu }}_{k-1}^{\left(1\right)},P_{k-1}^{\left(1\right)}\right)1_{S_{k-1}^{\left(j,m\right)}}\left(H_{k-1}^{\left(j\right)}{\underline{x}}_{k-1}^{\mathrm{mot}}+{\underline{d}}_{k-1}^{\left(j\right)}\right) \end{array}$$

follow from the Gaussian forward/backward projection of Appendix B, now indexed by (j,m) and applied only to the living prior (s=1), with auxiliary variable ${\underline{\xi }}^{\left(j\right)}=H_{k-1}^{\left(j\right)}{\underline{x}}_{k-1}^{\mathrm{mot}}+{\underline{d}}_{k-1}^{\left(j\right)}$. This yields $w_{j,m}$, ${\underline{\mu }}_{\underline{x},\left(j,m\right)}$, and $P_{\underline{x},\left(j,m\right)}$ calculated using the formulas for the moments of bivariately truncated Gaussians from [60].

### Appendix C.2. Final Composed Moments

From Equation (A2), the soft-weighted ejection contribution of the living prior is

$$\begin{array}{cc} M_{0}^{\mathrm{ej}} & ={\tilde{\pi }}_{k-1}^{\left(1\right)}\sum_{j=1}^{N_{A}}\sum_{m\in \Omega_{\mathrm{in}}}p_{\mathrm{eject},m}\;w_{j,m}\;,\\ M_{1}^{\mathrm{ej}} & ={\tilde{\pi }}_{k-1}^{\left(1\right)}\sum_{j=1}^{N_{A}}\sum_{m\in \Omega_{\mathrm{in}}}p_{\mathrm{eject},m}\;w_{j,m}\;{\underline{\mu }}_{\underline{x},\left(j,m\right)}\;,\\ M_{2}^{\mathrm{ej}} & ={\tilde{\pi }}_{k-1}^{\left(1\right)}\sum_{j=1}^{N_{A}}\sum_{m\in \Omega_{\mathrm{in}}}p_{\mathrm{eject},m}\;w_{j,m}\left[P_{\underline{x},\left(j,m\right)}+{\underline{\mu }}_{\underline{x},\left(j,m\right)}{\left({\underline{\mu }}_{\underline{x},\left(j,m\right)}\right)}^{⊤}\right]\;. \end{array}$$

The unnormalized moments for mode r=0 are then obtained by adding the full prior of mode s=0 to the soft-weighted ejection contribution, that is,

$$\begin{array}{cc} M_{0}^{\left(r=0\right)} & ={\tilde{\pi }}_{k-1}^{\left(0\right)}+M_{0}^{\mathrm{ej}}\;,\\ M_{1}^{\left(r=0\right)} & ={\tilde{\pi }}_{k-1}^{\left(0\right)}{\underline{\mu }}_{k-1}^{\left(0\right)}+M_{1}^{\mathrm{ej}}\;,\\ M_{2}^{\left(r=0\right)} & ={\tilde{\pi }}_{k-1}^{\left(0\right)}\left[P_{k-1}^{\left(0\right)}+{\underline{\mu }}_{k-1}^{\left(0\right)}{\left({\underline{\mu }}_{k-1}^{\left(0\right)}\right)}^{⊤}\right]+M_{2}^{\mathrm{ej}}\;. \end{array}$$

Mode r=1 receives only the surviving mass of the living prior. Since $q^{\left(0\right)}+q^{\left(1\right)}$ recovers the full prior mixture, the complementary property of Appendix B remains valid. Equivalently, $M_{q}^{\left(r=1\right)}=M_{q,\mathrm{prior}}^{\left(s=1\right)}-M_{q}^{\mathrm{ej}}$, where $M_{q,\mathrm{prior}}^{\left(s=1\right)}$ is the s=1 summand of $M_{q,\mathrm{prior}}$. Reconstruction of ${\tilde{\pi }}_{k}^{\left(r\right)}$, ${\tilde{\underline{\mu }}}_{k-1}^{\left(r\right)}$, and ${\tilde{P}}_{k-1}^{\left(r\right)}$ from $M_{q}^{\left(r\right)}$ proceeds identically to Appendix B.

## Appendix D. Numerical Verification and Sensitivity Analysis of the ADF-Inspired Prediction

We numerically verify the ADF-inspired prediction scheme (Section 5.3) against Monte Carlo under realistic tracking uncertainty. In particular, we check that predicted costs stay close enough to Monte Carlo not to corrupt sequence selection. We first compare predicted position densities after actuator contacts (qualitative, Appendix D.1), then the terminal costs that enter sequence selection (quantitative, Appendix D.2). Additionally, we perform a limited model-mismatch sensitivity analysis to exemplarily assess the robustness of the ADF-inspired prediction scheme to model mismatch and miscalibrated tracking uncertainty (quantitative, Appendix D.3).

Unlike the simulation evaluation in Section 7.1, which used ground-truth particle states with artificial uncertainty, all three checks start from predictive-tracking beliefs. These estimated particle states are propagated by ADF and by a Monte Carlo implementation of the system model from Section 5.2. All other settings match Section 7.1 unless stated otherwise; the parameters relevant to reproducibility are listed in Table A3. For the numerical verification (Appendix D.1 and Appendix D.2), the only modeling difference is particle–actuator arrival: Monte Carlo replaces the Gauß–Taylor approximation by a finer sampling interval *T* (50 times smaller than for ADF) with linear interpolation between the samples before and after arrival. The resulting Monte Carlo distributions therefore serve as reference predictions for the nominal system model. For the sensitivity analysis (Appendix D.3), the ADF prediction remains at its nominal model settings, whereas selected parameters of the Monte Carlo truth model are varied one at a time. The resulting Monte Carlo costs are then compared with the unchanged ADF-predicted terminal costs.

### Appendix D.1. Qualitative Verification: Position Densities After Actuator Contacts

Figure A1 and Figure A2 show predicted position densities at stages $n^{★}-1,\ldots ,n^{★}+3$ around an actuator contact at stage $n^{★}$ for two example particles (Monte Carlo: $10^{4}$ samples each). Both particles were selected as partial hits, i.e., with existence probability near 0.5 in at least one post-contact stage, so that the two existence modes do not coincide. In Figure A1, the particle is first contacted by an actuator at x = 465 mm in stage $n^{★}=2$, covering part of its arrival-time distribution, and then by a second actuator at x = 585 mm in stage $n^{★}+1$ (from stage $n^{★}+1$ onward, the Monte Carlo living-mode density ex=1 is poorly resolved because only few samples remain). In Figure A2, the particle is partially hit by an actuator at x=585 mm spanning y∈35 mmto 44 mm and thus part of its arrival-location distribution.

Despite these challenging partial-hit cases, the ADF with Gauß–Taylor arrival approximation covers the Monte Carlo densities well. Deviations appear mainly immediately after contact, where Monte Carlo densities often show sharp cut-offs from the finite actuator width carving a bounded impact region out of the incoming density. A few stages later, process noise restores near-Gaussian shapes that the ADF overlays closely.

Approximation quality also depends on the width of the predictive distribution relative to actuator duration and width: for broad beliefs, carving out a bounded impact region can leave residual multimodality (not shown here). The main driver of such uncertainty is the number of associated measurements. However, those high-uncertainty particles by design contribute little to the open-loop terminal cost $J_{k}$ (cf. Section 5.4), as confirmed quantitatively below. The stochastic cost-to-go therefore down-weights poorly observed tracks—a clear advantage over a deterministic MPC cost on a single nominal trajectory.

### Appendix D.2. Quantitative Verification: Cost-to-Go Comparison

We next evaluate, for each candidate sequence ${\underline{u}}_{k,0:N-1}^{\left(i\right)}$ in the MTCR enumeration, the cost-to-go $J_{k}$ (Section 5.4) via the ADF forward chain and, independently, via a Monte Carlo estimate of the same $J_{k}$ with 1000 samples. Writing $N_{P}$ for the number of tracked particles at time step *k*, the signed per-particle cost gap is

$$\begin{array}{c} \delta_{k}\left({\underline{u}}_{k,0:N-1}^{\left(i\right)}\right)=\frac{J_{k}^{\mathrm{MC}}\left({\underline{u}}_{k,0:N-1}^{\left(i\right)}\right)-J_{k}^{\mathrm{ADF}}\left({\underline{u}}_{k,0:N-1}^{\left(i\right)}\right)}{N_{P}}\in \left[-1,1\right]\;. \end{array}$$

Positive $\delta_{k}$ means ADF underestimates the Monte Carlo cost (ADF optimistic for minimization); negative $\delta_{k}$ means it overestimates it. Let ${\underline{u}}_{k,0:N-1}^{★,\mathrm{ADF}}={\arg\min}_{i}J_{k}^{\mathrm{ADF}}\left({\underline{u}}_{k,0:N-1}^{\left(i\right)}\right)$ and ${\underline{u}}_{k,0:N-1}^{★,\mathrm{MC}}={\arg\min}_{i}J_{k}^{\mathrm{MC}}\left({\underline{u}}_{k,0:N-1}^{\left(i\right)}\right)$. Whenever these argmins differ, the associated normalized Monte Carlo regret is

$$\begin{array}{c} R_{k}=\frac{J_{k}^{\mathrm{MC}}\left({\underline{u}}_{k,0:N-1}^{★,\mathrm{ADF}}\right)-J_{k}^{\mathrm{MC}}\left({\underline{u}}_{k,0:N-1}^{★,\mathrm{MC}}\right)}{N_{P}}\in \left[0,1\right]\;, \end{array}$$

i.e., the excess Monte Carlo cost of following the ADF-selected sequence instead of the Monte Carlo optimum. The ratio of controller calls with disagreeing decisions to the total number of controller calls is denoted by $r_{\mathrm{disagree}}$.

The run uses 200 controller calls, and 542 sequence evaluations (mean 2.71 sequences per call; range 1–4), with Monte Carlo reference costs based on 1000 samples per sequence. Table A1 pools $\delta_{k}$ over all sequence evaluations and summarizes $R_{k}$ per call. It summarizes the results for the current evaluation in its first row (the remaining rows subsume the results of Appendix D.3). $\delta_{k}$ is negative (ADF overestimates) in 56.8% of samples and positive (ADF underestimates) in 41.7%. Monte Carlo and ADF argmins differ in 76/200 calls (38.0%). Per-particle gaps and regrets remain small relative to the O(1) terminal penalties, so ADF-guided sequence selection stays close to the Monte Carlo-optimal choice.

**Table A1:** Empirical distribution of per-sequence cost gaps $\delta_{k}$ (pooled over all sequence evaluations) and Monte Carlo regret $R_{k}$ for calls in which the selected sequences disagree. The regret statistics are therefore conditional on a decision disagreement; $r_{\mathrm{disagree}}$ reports the fraction of all calls with such a disagreement. The first row presents the results for the numerical verification of the ADF-inspired sequence selection vs. Monte Carlo in Appendix D.2 (reference case); the remaining rows present the results for the limited model-mismatch sensitivity analysis in Appendix D.3. For ease of comparison, the parameter values of the reference case are shown in parentheses within each heading. Reported statistics include the 5% and 95% quantiles, $q_{0.05}$ and $q_{0.95}$, respectively.

	$q_{0.05}\left(\delta_{k}\right)$	Median $\delta_{k}$	$q_{0.95}\left(\delta_{k}\right)$	Median $R_{k}$	$q_{0.95}\left(R_{k}\right)$	$r_{\mathrm{disagree}}$
Monte Carlo vs. ADF, no mismatch (Reference case)						
	−4.0 · 10^−3^	−9.1 · 10^−5^	9.1 · 10^−4^	5.8 · 10^−4^	2.5 · 10^−3^	38.0
Ejection probabilities $\left(p_{\mathrm{eject},\mathrm{HIT}},p_{\mathrm{eject},\mathrm{UP}},p_{\mathrm{eject},\mathrm{DOWN}}\right)$ for accept/reject class (Ref.: (1.0,1.0,1.0)/(0.95,0.0,0.0))						
(1.0,1.0,1.0)/(0.9,0.35,0.05)	−5.7 · 10^−4^	4.8 · 10^−3^	1.1 · 10^−2^	8.8 · 10^−4^	3.0 · 10^−3^	35.0
(1.0,1.0,1.0)/(0.8,0.7,0.1)	0.0	1.5 · 10^−2^	3.5 · 10^−2^	1.1 · 10^−3^	2.4 · 10^−3^	39.0
(0.8,0.7,0.1)/(0.8,0.7,0.1)	−3.1 · 10^−2^	1.4 · 10^−2^	3.3 · 10^−2^	9.2 · 10^−4^	3.0 · 10^−3^	38.0
Phase durations $\left(t_{\mathrm{activate}}^{\mathrm{act}},t_{\mathrm{up}}^{\mathrm{act}},t_{\mathrm{hit}}^{\mathrm{act}},t_{\mathrm{down}}^{\mathrm{act}},t_{\mathrm{reset}}^{\mathrm{act}}\right)$ (Ref.: (10.0,0.0,2.5,5.0,5.0))						
(10.0,0.0,2.5,7.5,2.5)	−3.6 · 10^−3^	−5.0 · 10^−5^	−1.7 · 10^−3^	−5.0 · 10^−4^	−2.0 · 10^−3^	34.5
(7.5,2.5,2.5,7.5,2.5)	−3.4 · 10^−3^	−6.4 · 10^−5^	−1.8 · 10^−2^	−6.1 · 10^−4^	−1.2 · 10^−2^	37
(12.5,0.0,2.5,0.0,7.5)	−2.5 · 10^−3^	8.6 · 10^−2^	2.3 · 10^−1^	2.8 · 10^−4^	3.7 · 10^−3^	48.0
Process-noise PSD in the x- and y-directions (Ref.: $10^{-7},10^{-7}$)						
$\left(10^{-3},10^{-3}\right)$	0.0	6.6 · 10^−2^	1.5 · 10^−1^	5.6 · 10^−3^	2.5 · 10^−2^	14.5
$\left(10^{-6},10^{-6}\right)$	−3.1 · 10^−3^	2.1 · 10^−4^	6.3 · 10^−3^	5.0 · 10^−4^	1.9 · 10^−3^	26.5
$\left(10^{-10},10^{-10}\right)$	−4.4 · 10^−3^	−1.3 · 10^−4^	8.4 · 10^−4^	5.6 · 10^−4^	1.7 · 10^−3^	34.5
Initial covariance scale (Ref.: 1)						
0.1	−1.0 · 10^−2^	−2.3 · 10^−4^	1.1 · 10^−3^	6.4 · 10^−4^	1.8 · 10^−3^	45.5
10	−1.3 · 10^−3^	1.1 · 10^−2^	3.4 · 10^−2^	7.6 · 10^−3^	2.4 · 10^−2^	8.0
100	0.0	5.5 · 10^−2^	1.2 · 10^−1^	6.1 · 10^−3^	1.5 · 10^−2^	11.5

### Appendix D.3. Model-Mismatch Sensitivity Analysis

The preceding verification concerns agreement between the ADF approximation and Monte Carlo for the designed system model. It does not, however, establish robustness to an incorrectly specified system model. We therefore perform a limited model-mismatch sensitivity analysis that examines selected model parameters and the state uncertainty supplied by the tracker. The analysis is intended to characterize the effect of these parameters, rather than to identify the statistically supported validity range of the model.

The ADF-inspired prediction scheme uses the system model from the previous numerical verification, whereas the Monte Carlo reference in this analysis uses an independently configured “truth” model. We vary four components around their nominal settings: (i) phase-dependent ejection probabilities, (ii) the five actuator-phase durations, (iii) the process-noise covariance of the particle motion (fully specified by its power spectral density (PSD), see e.g., [48]), and (iv) the initial particle-state covariance supplied to the prediction model. The initial covariance is included because it depends strongly on the tuning and initialization of the tracker. We do not vary actuator geometry, including actuator position and width, because it is known comparatively accurately from the mechanical design. We also retain the assumptions that actuator states, estimated particle-state means, and particle classes are correct on average. A systematic error in these quantities would shift the incoming particle and actuator states or the intended actuator actions and would therefore constitute a tracking, classification, or state-estimation error rather than the model-prediction mismatch studied here. The comparison cases vary one component at a time while all other settings remain fixed. Evaluation settings and metrics are the same as in Appendix D.2. Table A1 lists the comparison cases and their results, with the reference case (no mismatch, as discussed in Appendix D.2) in the first row.

The two strongest ejection-probability perturbations produce positive median cost gaps of approximately 1.5 · 10^−2^ and 1.4 · 10^−2^, respectively. The third phase-duration setting (possibly because it shifts the beginning of the HIT phase) and the high process-noise setting are the most pronounced sources of positive cost gaps, with median gaps of approximately 8.6 · 10^−2^ and 6.6 · 10^−2^, respectively. The covariance-scale experiments show a similar transition: scales 10 and 100 yield positive median gaps, whereas the smaller scale 0.1 has a median gap close to zero.

These positive gaps indicate that, in these regimes, the ADF systematically underestimates the Monte Carlo cost. A plausible mechanism is that larger uncertainty makes the nonlinear, bounded contact dynamics more influential: the resulting predictive distribution can become skewed or truncated near contact boundaries, whereas a moment-matched Gaussian representation retains only its first two moments. This interpretation is qualitative; the experiments do not isolate which non-Gaussian feature dominates. The disagreement rate can nevertheless decrease in these regimes because a systematic cost shift need not change the ranking of candidate sequences. The conditional regret, however, is clearly larger for the high process-noise and enlarged-covariance settings. The results therefore identify uncertainty-model mismatch, especially inflated process noise PSD and tracker covariance, as the more stressful regimes. Conversely, the tested variations in ejection probability and actuator timing affect the selected sequence less consistently and show relatively low regret.

## Appendix E. Ablation: Effect of Retaining the FAF Base Sequence

Table A2 compares closed-loop sorting metrics with and without appending the standalone FAF sequence to ${\left\{{\underline{u}}_{k,0:N-1}^{\left(i\right)}\right\}}_{i=1,2,\ldots }$ for the default tree size $N_{R}=N_{B}=2$ and $c_{\mathrm{acc}}=0.5$, under the same simulation settings as Table 1. Under medium mass flow the rates are essentially unchanged. Under high mass flow, retaining FAF slightly lowers TNR (and raises DNR), consistent with occasionally selecting the FAF sequence when it ranks better under $J_{k}$ while remaining weaker in closed-loop rejection. Because forced retention does not improve closed-loop accuracy, all reported simulation and laboratory results use MTCR without forcing the FAF sequence into ${\left\{{\underline{u}}_{k,0:N-1}^{\left(i\right)}\right\}}_{i=1,2,\ldots }$.

**Table A2:** Sorting results with and without retaining the standalone FAF sequence in the set of candidate control sequences ${\left\{{\underline{u}}_{k,0:N-1}^{\left(i\right)}\right\}}_{i=1,2,\ldots }$ (MTCR with $c_{\mathrm{acc}}=0.5$, $N_{R}=N_{B}=2$; simulation as in Section 7.1).

	Medium Mass Flow		High Mass Flow	
	TNR/TPR in %	DNR/DPR in %	TNR/TPR in %	DNR/DPR in %
Without FAF retention	93.3/96.2	6.2/3.5	91.2/91.2	8.3/8.1
With FAF retention	93.4/96.2	6.1/3.5	90.7/91.3	8.8/8.0

## Appendix F. Reproducibility Parameters

Table A3 tabulates the main parameters needed to reproduce the reported simulation results. Parameters used in the laboratory experiments deviate from the ones in the simulation evaluation and are documented in Section 7.3. Any laboratory parameters not listed there match the values in Table A3.

**Table A3:** Main reproducibility parameters for the simulation results. Parameters are grouped according to whether they belong to the MTCR controller, the particle, actuator, or contact model, the simulator, or the tracking filter. Tuples represent x- and y-components of the corresponding quantity.

Parameter	Entry	Value
MTCR		
Sampling interval	*T*	$t_{\mathrm{cycle}}^{\mathrm{act}}=22.5ms$
Horizon	*N*	10
Tree size limits	$N_{R}=N_{B}$	2 or 4
Cost weight	*c*	0.5 or 0.8
Retain FAF candidate	–	off (or on, only in Appendix E)
Particle model		
CV-process-noise PSD	–	$\left(1\;\cdot \;10^{-6},\;1\;\cdot \;10^{-6}\right)$ mm^2^/ms^3^
CV-process-noise PSD, only in Appendix D	–	$\left(1\;\cdot \;10^{-7},\;1\;\cdot \;10^{-7}\right)$ mm^2^/ms^3^
Actuator model		
Phase durations	$t_{\mathrm{activate}}^{\mathrm{act}},\;t_{\mathrm{UP}}^{\mathrm{act}},\;t_{\mathrm{HIT}}^{\mathrm{act}},\;t_{\mathrm{DOWN}}^{\mathrm{act}},\;t_{\mathrm{reset}}^{\mathrm{act}}$	10,0,2.5,5,5ms
Actuator width	$w^{\mathrm{act}}$	9 mm
Contact model		
Ejection probability, accept particle	$p_{\mathrm{eject},\mathrm{HIT}},p_{\mathrm{eject},\mathrm{UP}},p_{\mathrm{eject},\mathrm{DOWN}}$	1.0,1.0,1.0
Ejection probability, reject particle	$p_{\mathrm{eject},\mathrm{HIT}},p_{\mathrm{eject},\mathrm{UP}},p_{\mathrm{eject},\mathrm{DOWN}}$	0.95,0.0,0.0
Simulator		
Sampling interval	–	2.5 ms
Simulation steps per controller call	–	8
Number of particles (not used in Appendix D)	–	100,000
Number of controller calls (only used in Appendix D)	–	200
Reject fraction	–	0.5
Spawn position mean	–	(−2.5,62.5)mm
Spawn-position std. dev.	–	(0.0,375)mm
Initial velocity mean	–	(1.0,0.0)mm/ms
Initial-velocity std. dev.	–	(0.1,0.1)mm/ms
Spawn distribution (mean, std. dev.), medium mass flow	μ,σ	0.16,0.3
Spawn distribution (mean, std. dev.), high mass flow	μ,σ	0.32,0.6
CV-process-noise PSD	–	$\left(1\;\cdot \;10^{-7},\;1\;\cdot \;10^{-7}\right)$ mm^2^/ms^3^
Artificial state noise covariance (not used in Appendix D)	–	$1\;\cdot \;10^{-8}\;\cdot \;C^{\underline{w}}$
Measurement-noise variance (only used in Appendix D)	$C^{\underline{v}}$	0.001 mm^2^
Particle simulator seed	–	12345
Particle simulator seed, only in Appendix D.1	–	98765
Contact distance	$d_{\mathrm{contact}}$	1.25 mm
DISRUPT-impact region relative to actuator position (front, bottom, back, top)	–	−4.0,−5.0,0.0,5.0mm
Tracking, CV Kalman filter (only used in Appendix D)		
CV-process-noise PSD	–	$\left(1.0\;\cdot \;10^{-7},\;1.0\;\cdot \;10^{-7}\right)$ mm^2^/ms^3^
Measurement-noise variance	$C^{\underline{v}}$	0.001 mm^2^

## Appendix G. Particle Size Distribution

Figure A3 shows the class-wise particle-length distributions for a representative laboratory sorting run (900 particles; accept-to-reject ratio 2:1), as measured by the image-processing pipeline of the prototype grid sorter.

## Appendix H. A Lower-Bound Estimate of Pneumatic Sorting Energy

This appendix details the lower-bound estimate of electrical energy for the pneumatic reference sorter reported in Table 2. The goal is an optimistic yet practical lower bound on the energy required to generate the ejected compressed air and to drive the valves, deliberately omitting plant overheads that would only increase the pneumatic total.

### Appendix H.1. Given Quantities

For each of the ten pneumatic runs, we recorded the commanded cumulative nozzle-open time $t_{\mathrm{raw}}$ (sum of all valve activation durations in the run as specified by the nozzle bar controller). The supply overpressure was $p_{g}=3.5\mathrm{bar}$ (gauge). The FESTO MHJ10-S-0,35-MF fast-switching valves are reported with sonic conductance *C* = 0.4L/(s bar) and critical pressure ratio b=0.38. Catalog switching times at 4 bar are $t_{\mathrm{on}}=0.8\;\mathrm{ms}$ (opening) and $t_{\mathrm{off}}=0.4\;\mathrm{ms}$ (closing); coil supply powers are $P_{\mathrm{peak}}=7\;W$ (high-current phase) and $P_{\mathrm{hold}}=2\;W$ (low-current phase). The downstream passage including the nozzle throat is not narrower than the valve restriction, so the valve limits the flow.

### Appendix H.2. Assumed Quantities

Ambient pressure $p_{\mathrm{amb}}=1\mathrm{bar}$ (absolute) and air temperature $T_{0}=T_{\mathrm{ref}}=293\;K$ (20 °C) match the pneumatic standard reference atmosphere (ANR) defined in ISO 8778 ( 100 kPa, 20 °C, 65% relative humidity). Air is treated as an ideal gas with heat capacity ratio γ=1.4, specific heat capacity at constant pressure *cp* = 1005 J/(kg K), and ANR density $\rho_{0}=1.185\;\mathrm{kg}/m^{3}$ as specified for standard-volume flow in ISO 6358-1. Overall compressor efficiency is set to η=0.85 (high-efficiency industrial compressor). Each run ejects $N_{P,\mathrm{rej}}=300$ reject particles with on average $n_{\mathrm{noz}}=3$ nozzles per particle (particle size ≈ 11.5 mm versus 5.2 mm nozzle pitch, plus one safety nozzle), hence $N_{\mathrm{act}}=N_{P,\mathrm{rej}}\;n_{\mathrm{noz}}=900$ activations. The high-current electrical phase duration is taken as $t_{\mathrm{peak}}=3\;\mathrm{ms}$ per activation from the supply-current plot in the manufacturer’s catalogue.

### Appendix H.3. Compressed-Air Energy

Absolute supply pressure is $p_{0}=p_{g}+p_{\mathrm{amb}}$. According to ISO 6358-1, with $p_{\mathrm{amb}}/p_{0}\approx 0.222\leq b$, flow is choked and the standard-volume flow rate is

(A3) $$\begin{array}{c} {\dot{V}}_{N}=C\;p_{0}\sqrt{\frac{T_{\mathrm{ref}}}{T_{0}}}\;. \end{array}$$

The corresponding mass-flow rate is $\dot{m}=\left({\dot{V}}_{N}\times 10^{-3}\;m^{3}/L\right)\;\rho_{0}$. For adiabatic reversible (isentropic) compression of an ideal gas in a steady-flow compressor, the specific work input $w_{\mathrm{is}}$ equals the enthalpy rise $\Delta h=c_{p}\;\Delta T$. Eliminating the temperature rise ΔT via Poisson’s isentropic temperature–pressure relation yields

$$\begin{array}{c} w_{\mathrm{is}}=c_{p}\;T_{0}\left[{\left(\frac{p_{0}}{p_{\mathrm{amb}}}\right)}^{\frac{\gamma -1}{\gamma }}-1\right]\;. \end{array}$$

While a valve is fully open, the instantaneous compressor electrical power is $P_{\mathrm{el}}=\dot{m}\;w_{\mathrm{is}}/\eta$, so the compressor energy for a corrected open duration $t_{\mathrm{corr}}$ is $E_{\mathrm{air}}=P_{\mathrm{el}}\;t_{\mathrm{corr}}$. The correction

$$\begin{array}{c} t_{\mathrm{corr}}\approx t_{\mathrm{raw}}-N_{\mathrm{act}}\;t_{\mathrm{on}} \end{array}$$

accounts for the opening transient (full choked flow is not attributed during $t_{\mathrm{on}}$). After the command ends, air continues to escape for about $t_{\mathrm{off}}=0.4\;\mathrm{ms}$ while the valve closes; this post-close contribution is omitted, which keeps $t_{\mathrm{corr}}$—and thus $E_{\mathrm{air}}$—a lower bound.

### Appendix H.4. Valve Electrical Energy

Valve supply energy is computed from the catalogue peak/hold powers with the assumed peak duration,

$$\begin{array}{c} E_{\mathrm{valve}}=N_{\mathrm{act}}\;P_{\mathrm{peak}}\;t_{\mathrm{peak}}+P_{\mathrm{hold}}\;\left(t_{\mathrm{corr}}-N_{\mathrm{act}}\;t_{\mathrm{peak}}\right) \end{array}$$

for $t_{\mathrm{corr}}\geq N_{\mathrm{act}}\;t_{\mathrm{peak}}$. The run total is $E_{\mathrm{total}}=E_{\mathrm{air}}+E_{\mathrm{valve}}$.

### Appendix H.5. Numerical Summary

Table A4 lists mean ± sample standard deviation over the ten runs. Under the stated pressure and valve parameters, Equation (A3) gives ${\dot{V}}_{N}=1.80\;L/s$ (≈108 L/min) and $P_{\mathrm{el}}=397\;W$. Real plant consumption can be higher because idle compressor operation, leaks, distribution, and nozzle closing losses are omitted.

**Table A4:** Intermediate quantities of the pneumatic energy lower-bound estimate (ten runs; mean ± sample standard deviation).

Quantity	Mean ± std. dev.
Logged open time $t_{\mathrm{raw}}$	12,050 ± 380 ms
Corrected open time $t_{\mathrm{corr}}$	11,330 ± 380 ms
Standard air volume $V_{N}$	20.39 ± 0.68 L
Compressor energy $E_{\mathrm{air}}$	4495 ± 151 J
Valve electrical energy $E_{\mathrm{valve}}$	36.2 ± 0.8 J
Total $E_{\mathrm{total}}$	4531 ± 152 J

## Appendix I. Experimental Statistics

Table A5 reports the confirmatory hypothesis tests from Section 7. For the Jonckheere–Terpstra test of decreasing mean TPR over c∈{0.2,0.5,0.8}, one obtains p=0.044; the uncorrected pairwise TPR superiority *p*-values are 0.016 (c=0.2 vs. c=0.5), 0.687 (c=0.5 vs. c=0.8), and 0.075 (c=0.2 vs. c=0.8).

**Table A5:** Confirmatory experimental tests (five runs per controller). Mean differences ${\hat{\Delta }}_{\mathrm{TNR}}^{\left(i\right)}$ and ${\hat{\Delta }}_{\mathrm{TPR}}^{\left(i\right)}$ are in percentage points for i∈{A,B,C} and equal the mean of the first named controller in $H_{1}^{\left(i\right)}$ minus the mean of the reference (see Section 7).

Hyp.	${\hat{\Delta }}_{\mathrm{TNR}}^{\left(i\right)}$	${\hat{\Delta }}_{\mathrm{TPR}}^{\left(i\right)}$	$p_{\sup}^{\left(i\right)}$	$p_{\mathrm{ni}}^{\left(i\right)}$	$p^{\left(i\right)}$	$p_{\mathrm{Holm}}^{\left(i\right)}$
$H_{1}^{\left(A\right)}$	+2.78	−0.53	0.012	0.008	0.012	0.024
$H_{1}^{\left(B\right)}$	+0.65	+0.70	0.016	0.103	0.103	0.103
$H_{1}^{\left(C\right)}$	—	+1.23	0.004	—	0.004	0.012

Table A6 reports statistics for the experimental differences relative to pneumatic predictive tracking, including nominal 95% confidence sets. These statistics are descriptive only, since they do not account for multiple testing. Therefore, confirmatory statistical significance cannot be inferred from the reported statistics in Table A6 alone.

**Table A6:** Descriptive statistics for the experimental differences relative to pneumatic predictive tracking (not Holm-adjusted), in percentage points. Statistics include the mean difference, defined as the mean of the named controller minus the mean of the pneumatic reference, the Hodges–Lehmann (HL) estimate (controller minus pneumatic predictive tracking), and nominal 95% confidence sets obtained by grid-based numerical inversion of the corresponding two-sided permutation and Wilcoxon rank-sum tests using their exact randomization distributions. Since all computed confidence sets were compact, they are reported by their lower and upper endpoints.

Controller	Metric	Mean Difference	Permutation Confidence Set	HL Estimate	HL Confidence Set
FAF	TNR	+2.78	[+0.44,+5.39]	+2.33	[+0.45,+6.33]
FAF	TPR	−0.53	[−1.32,+0.33]	−0.50	[−1.32,+0.33]
MTCR, c=0.2	TNR	+0.65	[−2.76,+4.01]	−0.22	[−2.64,+4.78]
MTCR, c=0.2	TPR	+0.70	[+0.09,+1.33]	+0.83	[+0.17,+1.33]
MTCR, c=0.5	TNR	+0.25	[−2.23,+3.00]	−0.22	[−2.30,+3.66]
MTCR, c=0.5	TPR	+0.07	[−0.58,+0.68]	+0.00	[−0.50,+0.83]
MTCR, c=0.8	TNR	−0.62	[−3.27,+2.33]	−0.88	[−3.56,+2.33]
MTCR, c=0.8	TPR	+0.17	[−0.66,+0.99]	+0.02	[−0.66,+0.99]
